# *Legionella pneumophila* type II secretome reveals a polysaccharide deacetylase that impacts intracellular infection, biofilm formation, and resistance to polymyxin- and serum-mediated killing

**DOI:** 10.1128/mbio.01393-25

**Published:** 2025-06-20

**Authors:** Carlton O. Adams, Jackson A. Campbell, Beichang Zhang, Leanne Cleaver, Sarah B. Bier, Joshua Mayoral, Richard C. White, James A. Garnett, Nicholas P. Cianciotto

**Affiliations:** 1 Department of Microbiology and Immunology, Northwestern University Medical School, Chicago, Illinois, USA; 2 Centre for Host-Microbiome Interactions, Faculty of Dental, Oral & Craniofacial Sciences, King’s College London, London, United Kingdom; Texas Christian University, Fort Worth, Texas, USA

**Keywords:** *L. pneumophila*, T2SS, polysaccharide deacetylase, PdaA, lipopolysaccharide, intracellular infection, autoaggregation, biofilm formation, resistance to polymyxin B, serum-resistance

## Abstract

Prior analyses suggested that the type II secretion system (T2SS) of *Legionella pneumophila* secretes ≥47 proteins beyond its 26 known substrates. Upon examination of mutants of wild-type strain 130b that lack those exoproteins most conserved across the *Legionella* genus, we discovered that protein “06635” majorly promotes *L. pneumophila* replication within amoebae. Immunoblotting, proteomics, and whole-cell enzyme-linked immunosorbent assay (ELISA) confirmed that 06635 exists in culture supernatants and on the bacterial outer surface and does so in a T2SS-dependent manner. Bioinformatic analyses identified 06635 as a novel member of the carbohydrate esterase family 4, whose members deacetylate bacterial surface polysaccharides, peptidoglycan, chitins, and/or xylans. Given 06635’s T2SS-dependent secretion, low-level amino acid similarity to known peptidoglycan deacetylases, and the unaltered lysozyme resistance of a *06635* mutant, we pursued the hypothesis that 06635 deacetylates a polysaccharide on *L. pneumophila*’s surface. Supporting this, the *06635* mutant exhibited increased binding to both wheat germ agglutinin (i.e., more surface *N*-acetylglucosamine) and antibodies that recognize acetylated lipopolysaccharide (LPS). Nuclear magnetic resonance (NMR) analysis of isolated mutant vs wild-type LPS confirmed that 06635 promotes LPS deacetylation. Thus, we designated 06635 as PdaA, for polysaccharide deacetylase A. Compatible with its altered surface, the *pdaA* mutant showed greater autoaggregation, increased biofilm formation, and heightened sensitivity to both polymyxin and human serum. Thus, we hypothesize that, following its secretion via the T2SS, PdaA deacetylates LPS, and perhaps other moieties, impacting many significant processes. While defining PdaA, we identified many more putative substrates of the *L. pneumophila* T2SS, bringing the size of the T2SS output to approximately 120.

**IMPORTANCE:**

*Legionella pneumophila* is the principal cause of Legionnaires’ disease, an increasingly common form of pneumonia. Although prior work demonstrated that the bacterium utilizes its type II protein secretion system (T2SS) to survive in aquatic environments and to cause lung infection, the full scope and impact of this *Legionella* secretion system is still relatively underappreciated. By utilizing an expanded proteomic approach and testing newly made mutants in a wide range of assays, we have determined that the *L. pneumophila* type II secretome encompasses approximately 120 proteins, and among these proteins is a novel polysaccharide deacetylase (PdaA) that modulates the *L. pneumophila* surface and lipopolysaccharide, impacting intracellular infection, biofilm formation, and resistance to both antibiotics and human serum. Moreover, since T2SSs and homologs of PdaA were found in many other bacteria, our findings should also have implications for understanding other infectious diseases and environmental processes.

## INTRODUCTION

*Legionella pneumophila* is the main agent of Legionnaires’ disease, a form of pneumonia that is increasing in prevalence (1, 2). In its natural and human-made water habitats, this gram-negative bacterium persists free-floating, as a constituent of biofilms, and as an intracellular parasite of amoebae, especially *Acanthamoeba* and *Vermamoeba* (3–6). After being inhaled as a component of aerosolized water droplets (7), *L. pneumophila* grows primarily in lung macrophages, and this is followed by extracellular spread, inflammation, and tissue destruction (5, 8–11). There are >66 *Legionella* species beyond *L. pneumophila*, and where examined, these species are also capable of intracellular infection and biofilm formation (12, 13).

Many gram-negative bacteria, especially members of the γ-Proteobacteria, utilize the type II secretion system (T2SS) for infection of hosts and/or growth in the environment (14, 15). In T2SSs, proteins to be secreted are first moved across the inner membrane by the Sec or Tat translocon, and then, after gaining their tertiary form in the periplasm, enter the T2SS apparatus for transport across the outer membrane and delivery into the extracellular space (16, 17). Although most defined T2SS substrates function in the extracellular milieu, some can associate with the bacterial surface after their release from the T2SS apparatus (14, 18–22). We have shown that the T2SS is important for the environmental survival and virulence of *L. pneumophila* and previously identified 26 substrates of the *L. pneumophila* strain 130b T2SS, including degradative enzymes and novel proteins (19, 23–26). Many of these 26 proteins are distributed across the *Legionella* genus and likely originated in a common ancestor of *Legionella* and *Aquicella*, whereas others resemble proteins from other bacteria, amoebae, mimiviruses, or fungi and may have been acquired by horizontal gene transfer (23). The T2SS promotes *L. pneumophila* survival in the lungs, fosters growth in macrophages and epithelia, and dampens cytokine secretion from host cells (27–31). T2SS substrate ChiA enhances survival in the lungs, possibly by acting as a mucinase, whereas the ProA protease degrades tissue and immune factors (19, 32, 33). The T2SS also enhances environmental aspects of *L. pneumophila*, i.e., infection of amoebae, biofilm formation, planktonic survival, sliding motility, and iron acquisition (23, 25, 26, 34–38). For example, T2SS mutants are ≥100-fold impaired for infection of at least four genera of amoebae, and eight of the known substrates are needed for optimal infection of environmental hosts (23, 24, 39–45).

Although the known output of the *L. pneumophila* T2SS is among the largest defined (14, 15, 23), initial analysis of the genome database using SignalP and PSORTb predicted that there are many more than 26 T2SS substrates (32). By reviewing proteomic data generated by others, we learned that 47 of these predicted substrates occur in *L. pneumophila* supernatants (23, 46–48). Prompted by a screen for those predicted T2SS substrates that are most conserved in *Legionella*, we uncovered a novel polysaccharide deacetylase (to be named PdaA) that modulates the outer surface of *L. pneumophila,* resulting in enhanced growth in amoebae, heightened resistance to polymyxin and serum, but diminished biofilm formation.

## RESULTS

### Protein 06635 promotes infection of amoebae by *L. pneumophila*

**Fig 1:**
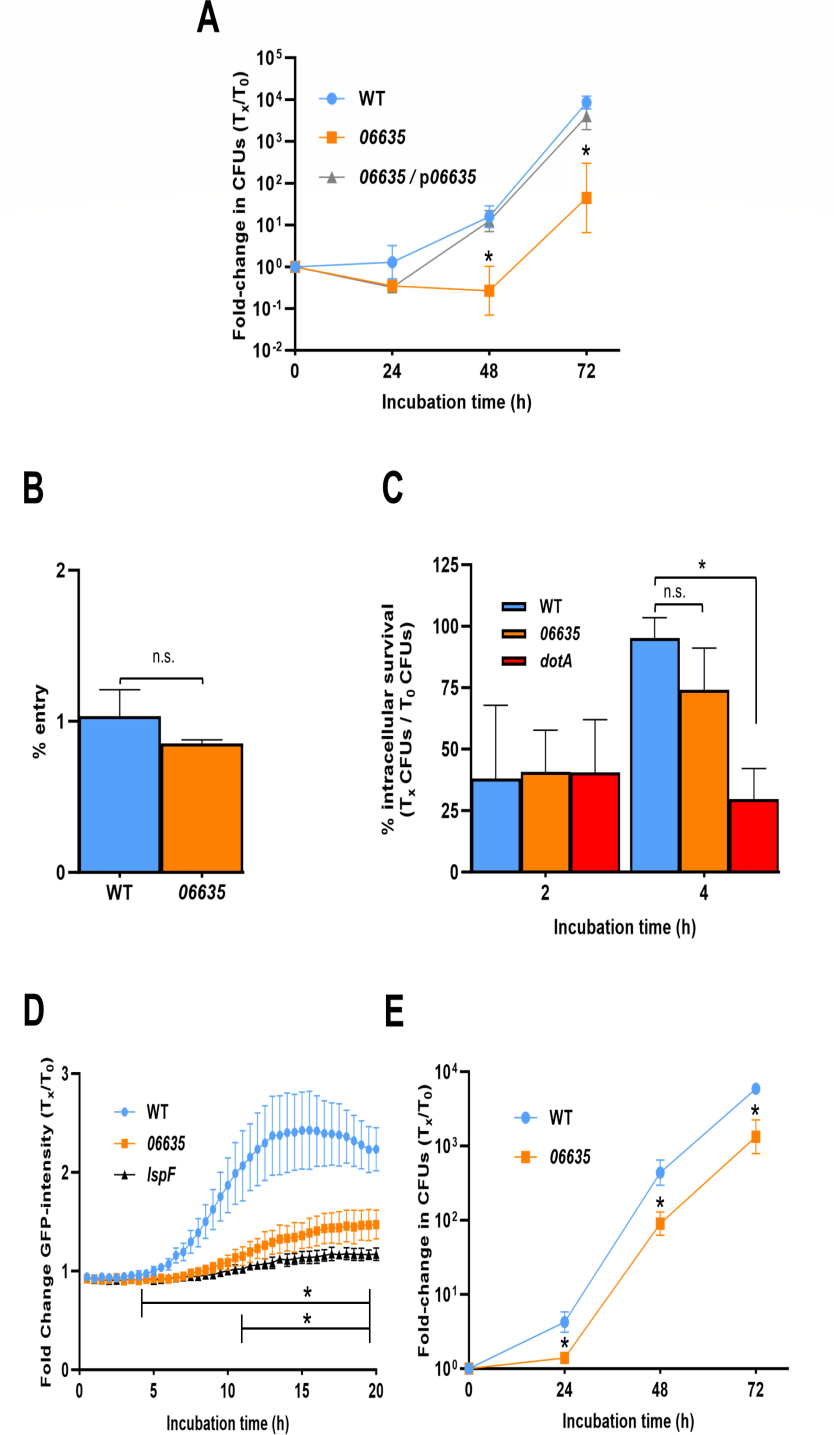
Effect of *06635* on *L. pneumophila* intracellular infection of amoebae. (**A**) Monolayers of *A. castellanii* were infected with WT strain 130b (WT), the *06635* mutant NU486 (*06635*), or NU486 containing plasmid-carried *06635* (*06635*/p*06635*) at an multiplicity of infection (MOI) = 0.1, and then immediately (i.e., *t* = 0) and at 24, 48, and 72 h post-inoculation, aliquots taken from the culture supernatants were assessed for bacterial numbers by plating for CFU on buffered charcoal yeast extract (BCYE) agar. Because *L. pneumophila* does not replicate in the medium, increases in CFU are due to bacterial growth in the amoebae. The values presented are the means and standard deviations from three technical replicates, and asterisks denote significant differences between the *06635* mutant and both WT and the complemented mutant; *P* < 0.05. (B and C) *A. castellanii* monolayers were infected with WT, *06635* mutant NU486, or *dotA* mutant NU428 at an MOI = 20. After centrifugation and a 1 h incubation to permit bacterial uptake by the amoebae, gentamicin was added to kill any remaining extracellular legionellae. After a final wash step, the amoebal cells were lysed either immediately (i.e., *t* = 0) (**B**) or at *t* = 0 and then 2 and 4 h later (**C**), and the numbers of released CFU were ascertained by plating. In panel **B**, entry is presented as the percentage of the inoculum that was protected from gentamicin treatment, and in panel **C**, early intracellular survival is presented as the ratio of recovered CFU at 2 and 4 h over the CFU recovered at time 0 × 100. The values presented are the means and standard deviations from three technical replicates, and the asterisk in panel **C** denotes a significant reduction for the *dotA* mutant; *P* < 0.05. (**D**) Monolayers of *A. castellanii* were infected were infected with green fluorescent protein (GFP)-expressing WT, *06635* mutant NU486, or *lspF* mutant NU275 at an MOI = 20. After centrifugation and a 1 h incubation to permit *L. pneumophila* uptake, gentamicin was added to kill any remaining extracellular bacteria. GFP fluorescence originating from the intracellular bacteria was monitored kinetically every 30 min for the next 20 h, and the fluorescence values obtained were normalized to the GFP signal at *t* = 0 following gentamicin treatment. The values presented are the means and standard deviations from six technical replicates. The asterisk above the upper horizontal line denotes the significant difference emerging between the two mutants and WT, whereas the asterisk above the lower horizontal line signifies when the *06635* mutant is different from the *lspF* mutant; *P* < 0.05. (**E**) Monolayers of *Vermamoeba vermiformis* were infected with WT strain 130b (WT) and the *06635* mutant NU486 (*06635*), and bacterial growth was monitored, analogously to what was described in panel **A**. Asterisks denote significant differences between the *06635* mutant and WT; *P* < 0.05. In panels B and C, n.s. = not significant. (A–E) All data presented are representative of the results obtained from at least three independent experiments.

We had ascertained that the more prevalent a *L. pneumophila* T2SS substrate is among other *Legionella* species, the more likely it was to be required for optimal infection of amoebae (23). Thus, we determined the genus-wide prevalences of the 47 proteins that were predicted by *in silico* analysis to be T2SS substrates and were previously detected in culture supernatants (23, 32, 46–48). Based on BLASTP searches, we pursued seven proteins encoded by ≥88% of the 66 *Legionella* species. In *L. pneumophila* strain 130b (26), these proteins’ locus tags are ABXK18_RS00420 (“*00420*”), ABXK18_RS06500 (“*06500*”), ABXK18_RS06635 (“*06635*”), ABXK18_RS08770 (“*08770*”), ABXK18_RS11870 (“*11870*”), ABXK18_RS12705 (“*12705*”), and ABXK18_RS13770 (“*13770*”). Whereas *06635* and *11870* had a 98% prevalence across the genus, *13770* and *08770* were 97% prevalent, *00420* 93%, *12705* 92%, and *06500* 88% (Table S1A). We introduced a deletion into each of the protein’s genes in wild-type (WT) 130b and tested the new mutants for infection of *Acanthamoeba castellanii*. Whereas mutants lacking *11870, 13770*, *08770, 00420, 12705*, or *06500* were not impaired (Fig. S1A), the *06635* mutant exhibited ~30- to 70-fold lower colony forming unit (CFU) recovery (Fig. 1A). A complemented *06635* mutant did not show this defect (Fig. 1A), confirming that the product of *06635* (i.e., “06635”) is required for optimal infection of *A. castellanii* by a WT strain of *L. pneumophila*.

Prior work showed that *lsp* mutants lacking the T2SS are normal for entry into *A. castellanii* and ensuing evasion of lysosomes (45). Compatible with this, when we assayed intracellular legionellae numbers immediately after inoculation of amoebae and then 2 and 4 h later, the *06635* mutant behaved like the WT strain (Fig. 1B and C). Thus, we posited that the *06635* mutant was impaired in the initiation of intracellular replication and/or the rate or final extent of growth. When we infected acanthamoebae with green fluorescent protein-expressing bacteria and examined increases in fluorescence in the intracellular compartment, the mutant, like an *lspF* T2SS mutant, displayed a slowed increase in fluorescence and a lower maximum fluorescence from 5 to 20 h of the primary round of infection (Fig. 1D). Together, these data indicated that 06635 promotes the replicative phase of intracellular infection by strain 130b.

Next, we tested the *06635* mutant for infection of *Vermamoeba vermiformis* and found it to be impaired ~4-fold (Fig. 1E), indicating that 06635 promotes infection of multiple amoebae. Other new mutants that had been tested in *A. castellanii* were found to grow normally in the vermamoebae (Fig. S1B). Though the *06635* mutant was impaired for growth in two types of amoebae, it replicated like WT did in buffered yeast extract (BYE) broth and in macrophage-like U937 cells (Fig. S2A and B), indicating that it does not have a generalized growth defect.

The Dot/Icm type IV secretion system (T4SS) has a critical role in intracellular infection by *L. pneumophila* (5, 9). However, the *06635* mutant appeared not to be lacking T4SS function. First, though defective for growth in the amoebae, the mutant was decidedly more infectious than *dot/icm* mutants, which exhibit impaired survival shortly after entering amoebae and are subsequently non-replicative (Fig. 1C; Fig. S3A) (5, 9). Second, the *06635* mutant grew in macrophages to the same level as WT did, whereas *dot/icm* mutants are completely impaired for growth in macrophages (5, 9). Finally, the *06635* mutant, like WT 130b, was very sensitive to NaCl, whereas *dot/icm* mutants exhibited marked salt resistance (Fig. S3B) (49–51).

Thus, 06635 promotes WT 130b replication in amoebae that are critical for the persistence of *L. pneumophila* in water systems. BLASTP searches identified genes/proteins corresponding to *06635*/06635 in 58/58 *L*. *pneumophila* strains examined (Table S1B), including the well-studied strains Corby, Lens, Paris, and Philadelphia-1 (52–54). For example, for Philadelphia-1 and its often-used derivatives, strains JR32 and Lp02 (52, 55), there is a gene (*lpg1993*) that shares 96% nucleotide identity with the *06635* gene and a protein (Lpg1993) that has 99% amino acid identity to the 06635 protein.

### Protein 06635 is a secreted substrate of the *L. pneumophila* T2SS

**Fig 2:**
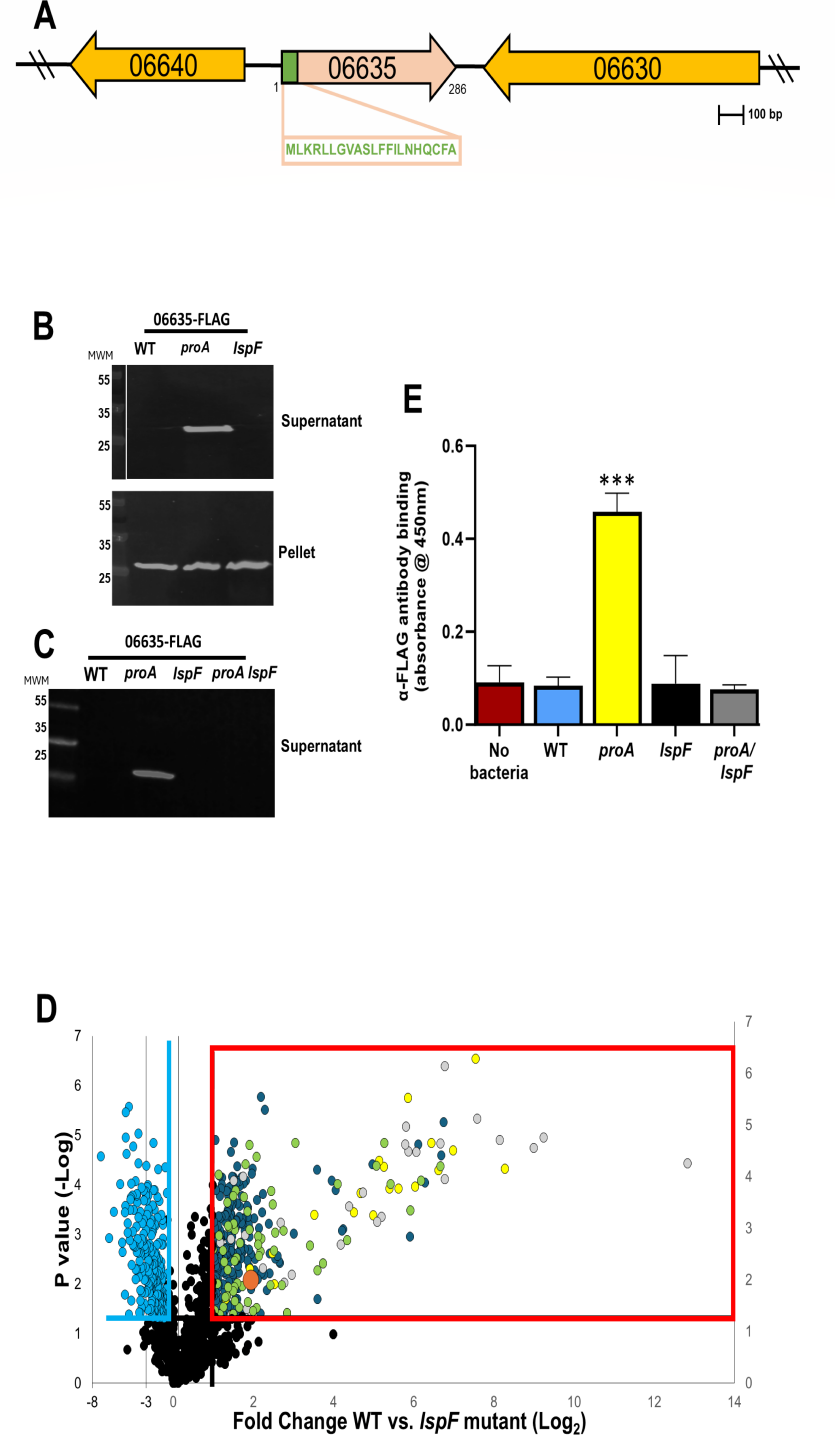
The 06635 gene locus and the T2SS-dependent secretion of the 06635 protein. (**A**) Depiction of the chromosomal locus of *L. pneumophila* strain 130b that encodes *06635*. In the map, the arrows indicate the relative length of each gene and the direction of their transcription. The *06630* gene (i.e., ABXK18_RS06630) downstream of *06635* is transcribed in the opposite direction and is annotated as a putative transglycosylase, whereas the *06640* gene (i.e., ABXK18_RS06640) upstream of *06635* is also divergently transcribed and is annotated as encoding a novel hypothetical protein. The box below the *06635* open reading frame (ORF) contains the amino acid sequence of the 06635 protein’s predicted signal sequence. A locus, like the one depicted here, occurs in the other *L. pneumophila* strains examined, including Philadelphia-1 and the Philadelphia-1 derivatives JR32 and Lp02. (**B**) WT strain 130b (WT), *proA* mutant AA200 (*proA*), and *lspF* mutant NU275 (*lspF*), each containing an isopropyl-beta-d-thiogalactopyranoside (IPTG)-inducible *06635* gene that encodes 06635 with a C-terminal FLAG tag (on p06635-FLAG1), were grown in BYE broth. Concentrated, cell-free culture supernatants were subjected to SDS-PAGE and then immunoblotted using anti-FLAG antibodies (top panel). Cell pellets obtained from the cultures were subjected to SDS-PAGE and the same immunoblot analysis (bottom panel). The top and bottom panels represent portions of the same gel, whose left-most lane contained pre-stained molecular weight markers (MWMs), with sizes in kDa. For the top panel, the image of the MWM lane was duplicated and (as the white space denotes) assembled next to the supernatant-containing lanes. (**C**) Supernatants obtained from wild-type strain 130b (WT), *proA* mutant AA200 (*proA*), *lspF* mutant NU275 (*lspF*), and *proA lspF* mutant NU493 (*proA lspF*), each carrying p06635-FLAG2, were subjected to immunoblot analysis, similarly to what was indicated in panel **B**. In both panels **B** and **C**, the images presented are the (only) portions of the blots showing proteins recognized by the antibodies, i.e., the 06635-FLAG protein. (**D**) Volcano plot depicting the differences in secreted proteins of *L. pneumophila* WT strain 130b (WT) vs *lspF* mutant NU275 (*lspF*) grown in BYE broth, as determined by proteomic analysis. The box outlined in red encompasses those proteins that were more abundant by at least twofold (*P* < 0.05) in the WT supernatant. The yellow dots denote proteins that had been previously confirmed to be T2SS substrates, the gray dots indicate proteins that had been previously detected in culture supernatants and predicted by *in silico* analysis to be T2SS substrates, the orange dot marks the protein 06635, and the green dots signify proteins that, based on *in silico* analysis, have a signal sequence, which is compatible with them being T2SS substrates. Light blue dots to the left of the vertical blue line denote proteins that were more abundant by at least twofold (*P* < 0.05) in the *lspF* mutant supernatant. Black dots indicate those proteins whose abundances were not different between WT and the *lspF* mutant. (**E**) WT strain 130b (WT), *proA* mutant AA200 (*proA*), *lspF* mutant NU275 (*lspF*), and *proA lspF* mutant NU493 (*proA lspF*), each expressing 06635-FLAG, were grown in BYE broth and then subjected to whole-cell enzyme-linked immunosorbent assay (ELISA) (five technical replicates/strain), where the presence of 06635-FLAG on the bacterial surface was measured by anti-FLAG-specific antibody binding, as detected by secondary antibody-associated absorbance at 450 nm. A phosphate buffered saline (PBS) buffer alone (no bacteria) was included as a control. The asterisks indicate greater surface expression of 06635-FLAG for the *proA* mutant relative to the other strains; ***, *P* < 0.0005. For panels **B**, **C**, and **E**, the results presented are representative of the outcome of at least three independent experiments. For panel **D**, the data are the results pooled from three biological replicates.

In strain 130b, *06635* is monocistronic (Fig. 2A). The gene is predicted to encode a 286-aa protein (Fig. 2A). The 06635 homologs from strains Corby, Lens, and Paris were also annotated as being 286 aa in length, whereas the 06635 homolog from strain Philadelphila-1 was annotated as being 292 aa, presumably based on a decision (here but not in the other cases) to utilize a rare start codon (TTG) that occurs upstream of a standard ATG start. As alluded to above, the 06635 protein has a 21-aa N-terminal signal sequence (Fig. 2A), and compatible with predictions obtained using PSORTb, it was previously seen in the culture supernatants of strain JR32, a streptomycin-resistant derivative of strain Philadelphia-1 (23, 32, 48, 55, 56). Thus, we made a C-terminal FLAG-tagged version of 06635, introduced it into WT and T2SS mutant *L. pneumophila*, and then examined culture supernatants for the presence of 06635 using immunoblot analysis, analogous to past studies (26, 57–60). Initially, we did not observe 06635 in WT 130b supernatants (Fig. 2B, top). We posited that 06635 or its FLAG-containing portion might have been degraded by ProA, the T2SS-dependent protease that can act on other exoproteins (10, 23, 26). Indeed, when we analyzed supernatants from a *proA* mutant (44), secreted 06635-FLAG was readily detected (Fig. 2B, top). The protein was not seen in supernatants of the *lspF* mutant lacking the T2SS (Fig. 2B, top), implying that 06635 is T2SS-dependent. When cell pellets derived from these samples were subjected to immunoblotting, all had similar levels of 06635 (Fig. 2B, bottom), affirming that the absence of 06635 in WT and *lspF* mutant supernatants was not due to an unforeseen lack of gene expression. For added proof of 06635’s T2SS dependency, we made a *proA lspF* mutant and found that it lacked 06635 in its supernatants unlike the *proA* mutant (Fig. 2C).

To affirm the T2SS dependency of 06635 by an alternative approach, we grew WT and the *lspF* mutant in BYE broth and then examined their culture supernatants by proteomics. Twenty of the 26 previously defined T2SS substrates were (2.5- to 310-fold) more prominent in the WT sample (Fig. 2D; Table S2). Moreover, 29 of the 47 proteins that had been predicted to be secreted and found before in *L. pneumophila* supernatants (23) were enriched (2.3- to 7,320-fold) in the WT sample, confirming that these 29 are linked to the T2SS (Fig. 2D; Table 1). However, importantly for the focus of the present study, among this group of 29 was 06635 (Fig. 2D; Table 1), bolstering the conclusion garnered from the immunoblot analysis. The increased presence of 06635 in WT samples (i.e., 3.9-fold) was greater than or like that of many of the other T2SS substrates. The WT T2SS+ sample was also enriched for 65 more proteins that had an N-terminal signal sequence (Fig. 2D; Table 2). Table S3 contains the full data set of proteins detected in the samples. Thus, this proteomic analysis (i) confirmed that 06635 is secreted via the T2SS and (ii) identified 94 new putative T2SS substrates.

**TABLE 1:** Previously detected exoproteins that are now confirmed as being T2SS substrates^*a*^

ORF^*b*^	Protein/predicted protein, annotation	Fold increase in WT supernatants over *lspF* mutant supernatants
00985	Neutral LasB-like metalloproteinase	7,320.4
12245	Choloylglycine hydrolase	608.3
11405	Ecto-ATP diphosphohydrolase II	512.5
05010	Novel protein	284.5
04070	Peptidase	192.6
15635	LvrE with DUF 1566	110.2
08375	M64 family IgA Peptidase	109.9
02545	5-Nucleotidase	102.2
07120	D-alanyl-D-alanine carboxypeptidase	67.3
03825	Novel protein	58.4
01835	Tyrosine phosphatase II protein LppA	56.2
11475	Lipase-like protein	55.4
10700	Leucyl aminopeptidase precursor	36.7
06500	Serine metalloprotease	34.0
03000	Phosphoesterase LegS1	26.8
00420	ATP-dependent zinc protease	21.0
05025	Bifunctional chitinase	18.2
10130	Novel protein	7.8
04875	Novel protein	6.9
08480	YceI-like domain-containing protein	6.4
04525	Novel protein	4.7
06635	Polysaccharide deacetylase	3.9
14365	Novel protein	3.6
09570	Novel protein	3.5
12725	IcmL-like protein	3.4
08470	YceI-like domain-containing protein	2.9
00335	Novel protein	2.7
14725	Eukaryotic-like protein with DUF 3421	2.7
02905	Metallopeptidase PepO	2.3

^*a*^ By using SignalP 6.0, all were re-confirmed as having an N-terminal signal sequence.

^*b*^ ORF, open reading frame.

**TABLE 2:** Signal sequence-containing proteins that are now detected in WT culture supernatants and to a lesser degree in T2SS mutant supernatants^*a*^

ORF^*b*^	Protein/predicted protein, annotation	Fold increase in WT supernatants over *lspF* mutant supernatants
08370	Glutaminase domain-containing protein	101.8
07015	Serine kinase	72.9
00750	Neutral metalloproteinase	60.8
03880	Alkaline phosphatase	43.0
11000	Alpha/beta hydrolase	38.5
10520	Glycosyl hydrolase family 3	33.8
09950	TPR repeat protein	20.3
10290	Proteinase inhibitor I42 chagasin protein	17.2
06855	Novel protein	13.3
11905	Novel protein	12.1
07145	Ectonucleoside triphosphate diphosphohydrolase I	10.6
08005	FecR domain-containing protein	8.3
03980	Cephalosporinase	7.2
00390	Conserved domain protein	6.7
02155	DUF 547-containing protein	6.6
02500	DotA	5.8
13140	Cupin domain-containing protein	5.7
11200	Outer membrane lipoprotein	5.6
00245	IcmL-like protein	5.4
04375	Superoxide dismutase [Cu-Zn] SodC	5.4
13095	Polysaccharide deacetylase	4.6
12645	Secreted lipoprotein	4.5
00225	Cytochrome c4	4.4
08855	3-Nucleotidase / nuclease	4.4
02485	IcmX (IcmY)	4.4
02105	L-sorbosone dehydrogenase	4.3
00725	Hemin-binding protein Hbp	4.2
07625	Novel protein	4.0
03975	Novel protein	4.0
05435	Chemiosmotic efflux system protein B	3.8
02555	DotC	3.7
12915	Hypothetical exported protein	3.7
04700	Novel protein	3.6
03230	Endolytic transglycosylase	3.5
09675	Transcriptional regulatory protein	3.4
07520	29 kDa immunogenic protein	3.3
12605	Beta-barrel domain-containing protein	3.2
03050	Novel protein	3.1
12575	Hypothetical signal peptide protein	3.0
00320	Peptide methionine sulfoxide reductase MsrA	3.0
02560	DotD	2.9
08980	Fimbrial biogenesis protein PilF	2.9
00900	Lipoprotein	2.9
07250	Apolipoprotein A1/A4/E	2.9
07225	Novel protein	2.9
12560	17 kDa common antigen	2.9
08140	16 kDa immunogenic protein	2.8
13360	Type II/III secretion system protein	2.8
08465	Signal peptide protein	2.8
08935	Arginine 3rd transport system protein artJ	2.6
01680	Adhesin head GIN domain-containing protein	2.6
14000	LphA (DotK)	2.6
11935	Cytochrome c type biogenesis protein	2.6
10075	SPOR domain-containing protein	2.6
10790	Putrescine-binding protein	2.5
10940	Novel protein	2.5
10315	YHS domain-containing protein	2.4
12610	Beta-barrel domain-containing protein	2.4
09185	Acyltransferase	2.4
07885	Outer membrane lipoprotein carrier protein	2.2
07645	Long-chain fatty acid transporter	2.2
12290	Outer membrane protein MIP	2.2
13530	Outer membrane lipoprotein LolB	2.2
07485	Thiol:disulfide interchange protein dsbA	2.1
15250	Catalase-peroxidase two katG2	2.0

^*a*^ By using SignalP 6.0, all were identified as having an N-terminal signal sequence.

^*b*^ ORF, open reading frame.

Since some T2SS substrates are linked to the bacterial outer surface in addition to being released into the extracellular milieu (19, 23, 25), we performed whole-cell enzyme-linked immunosorbent assays (ELISAs) to discern if 06635 is also present on the *Legionella* surface. As in culture supernatants, 06635-FLAG was not detected on the surface of WT but was readily detected on the surface of the *proA* mutant (Fig. 2E), indicating that some of the secreted 06635 molecules can remain at the outer surface of *L. pneumophila*. However, surface-associated 06635-FLAG was lost for the *lspF* mutant and *proA lspF* mutant (Fig. 2E), confirming that surface localization of 06635 is T2SS-dependent.

In sum, 06635 is a bona fide T2SS-dependent exoprotein, based on (i) *in silico* analysis that predicted it to be secreted beyond the cell envelope, (ii) its presence in culture supernatants of two different strains of *L. pneumophila*, as observed by two different research groups, i.e., strain 130b (above) and strain JR32 (48), and (iii) its reduced presence in culture supernatants and on the outer surface of a T2SS mutant of WT 130b, as discerned by three methods.

### Protein 06635 is a novel member of the carbohydrate esterase 4 (CE-4) superfamily

**Fig 3:**
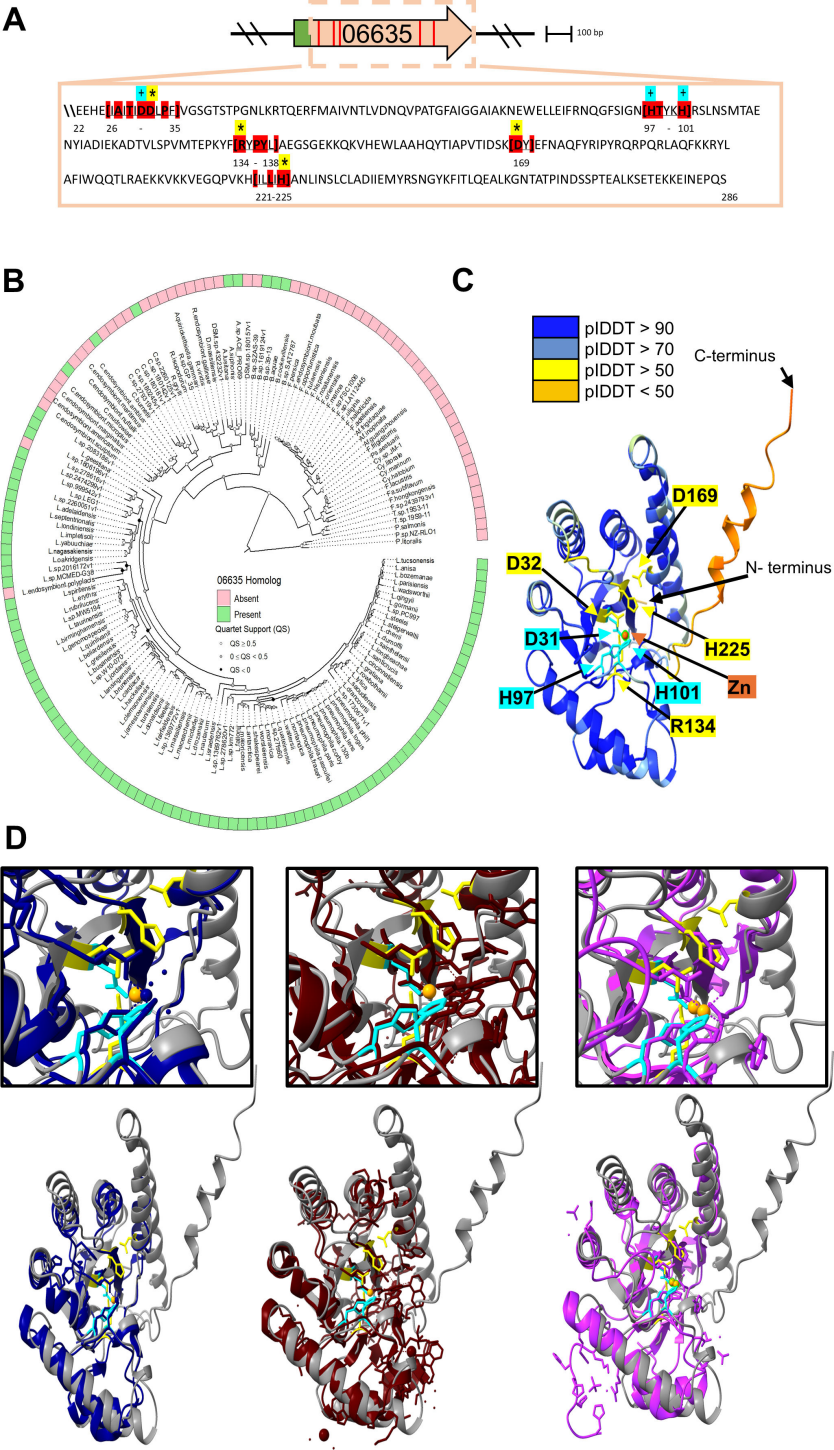
Signature residues, phylogenetic distribution, and predicted structure of the 06635 protein. (**A**) The box below the gene map contains the amino acid sequence of 06635, minus its N-terminal signal sequence. Residues highlighted in red are the conserved sequence motifs of the “NodB domain” of the CE-4 superfamily. A “+” symbol denotes those residues that typically contribute to the Zn-binding domain. The asterisks denote the residues that typically constitute the enzyme active site. (**B**) Distribution of genes encoding 06635 homologs within species of *Legionella* and related genera. (Center) A maximum-likelihood phylogenetic tree for unique species in the orders *Berkiellales*, *Coxiellales*, *Diplorickettsiales*, *DSM-16500*, *Francisellales, Legionellales*, and *Piscirickettsiales*. Scale, one amino acid substitution per site. (Outer ring) The presence of a gene corresponding to *06635* is marked by green squares, whereas the absence of a gene is denoted by pink squares, as determined using BLASTP. (**C**) Predicted 3-D structure of 06635 (without its N-terminal signal sequence) bound with Zn, as determined by AlphaFold 3. The predicted structure is color-coded in accordance with the levels of confidence (i.e., the predicted local distance difference test [plDDT] values) determined by the program (upper left). The protein’s N-terminus and C-terminus are denoted, as are the putative Zn-binding and active-site residues (in blue and yellow) and the location of the bound Zn (in red). (**D**) Alignments of the predicted 06635 structure with known structures, as analyzed using the DALI server. Presented here are the alignments of 06635 (dark gray) with the acetylxylan esterase of *C. subterraneus* (Protein Data Bank [PDB] ID: 7Y51) (dark blue, on left), giving a root mean square deviation (RMSD) = 2.1, Z = 21.3, and % identity = 27, the PG GlcNAc deacetylase of *B. cereus* (PDB 5O6Y) (dark brown, in center), exhibiting an RMSD = 2.2, Z = 20.6, and % identity = 25, and the chitin deacetylase of *Colletotrichum lindemuthianum* (PDB 2IW0) (dark purple, on right), giving an RMSD = 2.3, Z = 19.7, and % identity = 26. The boxes present an enlarged image of the D-H-H Zn-binding signature and active-site residues of the CE-4 family mapping to a region of alignment.

In the *L. pneumophila* genome, 06635 had been annotated as a polysaccharide deacetylase (23). We confirmed that 06635 has the conserved motifs, active-site residues, and Zn^2+^-binding residues of the CE-4 superfamily (61–65) (Fig. 3A). Characterized CE-4 members deacetylate one or more of the following: (i) the *N*-linked acetyl group from *N*-acetylglucosamine (GlcNAc) residues existing in chitin or bacterial peptidoglycan (PG), (ii) the *N*-linked acetyl group from *N*-acetylmuramic acid residues that can occur in PG, (iii) the *O*-linked acetyl group from *O*-acetylxylose residues in cellulose acetate and xylan, and (iv) the *N*-linked acetyl group from bacterial exopolysaccharides such as poly-β-1,6-*N*-acetylglucosamine (62, 64, 66–75). Thus, we did an expansive BLASTP search with the aim of identifying a defined homolog. Protein 06635 had its greatest level of relatedness (i.e., 47% aa identity, 88% coverage; *E* value = 3e−84) to a *L. pneumophila* hypothetical protein, e.g., protein 13095 of strain 130b. Like 06635, 13095 has a signal sequence and, from our proteomics, is secreted by the T2SS (Table 2). Aligning with our initial search (Table S1A), proteins highly related to 06635 existed in 90/92 *Legionella* genomes (Fig. 3B). Among the non-*Legionella* homologs, 06635 shared its greatest relatedness (i.e., 50% aa identity, 81% coverage; *E* = 4e−79) to a hypothetical polysaccharide deacetylase from *Coxiella burnetii*, agent of Q fever and a Legionellales member (Fig. 3B; Table S4A) (76). Other related proteins in the Legionellales were hypothetical proteins from *Aquicella siphonis* and *Berkiella aquae* (Fig. 3B; Table S4A) (76–78). The next set of “hits” (i.e., 36%–28% aa identity; *E* = 8e−37 to 2e−25) were all hypothetical polysaccharide deacetylases from a range of gram-negatives, including pathogenic *Stenotrophomonas maltophilia* (Table S4A) (79, 80). From BLASTP searches, 06635 had much less relatedness (i.e., *E* = 3e−10 to 3e−08) to known enzymes, i.e., the PG GlcNAc deacetylases of *Streptococcus pneumoniae*, *Bacillus cereus*, and *Mycobacterium tuberculosis* (63, 64, 81–84) and an acetylxylan esterase from *Caldanaerobacter subterraneus* (73) (Table S4B). Thus, 06635 appeared to be representative of a large but uncharacterized branch in the CE-4 superfamily.

We further analyzed 06635 using the structural prediction program AlphaFold (85) and obtained a high-confidence model with bound Zn^2+^ (interface predicted template modeling [ipTM] = 0.98; predicted template modeling [pTM] = 0.88) (Fig. 3C). The model aligned with three known structures, i.e., the above-noted acetylxylan esterase of *C. subterraneus* and PG deacetylase of *B. cereus*, and a chitin deacetylase from *Colletotrichum lindemuthianum* (86) (Fig. 3D). However, since these alignments had similar statistical outputs, they, like the BLASTP results, did not indicate what molecule is the substrate(s) of 06635.

Though 06635 had low-level relatedness to some gram-positive PG deacetylases, it did not appear to be simply a PG deacetylase. First, 06635 is a T2SS-dependent, secreted protein (and T2SS substrates are not described as being active until after they transit the outer membrane), whereas PG deacetylases of gram-negative bacteria remain in the cell (87). Second, 06635 appeared to have no similarity to any defined gram-negative PG deacetylase (87–90), i.e., there were no matches from BLASTP searches that had an *E* value below the threshold of 0.05. Third, unlike PG deacetylase mutants of other gram-negatives and gram-positives (63, 88, 89, 91–94), the *06635* mutant did not show increased sensitivity to lysozyme, whose target is PG (95–97) (Fig. S2C). Fourth, the *L. pneumophila* 130b genome revealed another protein, 08520, that did have compelling similarity to gram-negative PG deacetylases (Fig. S4A and B) and was not seen in supernatants. The fact that 08520 does not have a signal sequence is compatible with it being periplasmic, since gram- PG deacetylases are translocated by Sec-independent means (90).

Overall, our bioinformatic analyses, along with the T2SS-dependent secretion of 06635 to the *L. pneumophila* outer surface and into the extracellular milieu, suggested that 06635 deacetylates (i) a GlcNAc-containing polysaccharide on the *L. pneumophila* surface, (ii) a GlcNAc-containing polysaccharide in the environment, and/or (iii) another acetyl-containing substrate(s). It was recently reported that 06635’s equivalent in *L. pneumophila* strain Lp02 (i.e., protein Lpg1993) can deacetylate purified *Vibrio cholerae* PG *in vitro* (98). Also, an Lp02 mutant lacking Lpg1993 exhibited increased sensitivity to lysozyme and had ~15% deacetylated glucosamine in its isolated PG vs ~28% deacetylated glucosamine in the PG from parental Lp02 (98). Thus, it was concluded that Lpg1993 acts as a PG deacetylase. Other phenotypes, including increased resistance to NaCl, indicated that the Lp02 *lpg1993* mutant had an impaired Dot/Icm T4SS, helping explain its impaired intracellular infectivity (98). However, the cellular location(s) and possible extracellular nature of Lp02’s Lpg1993 were not experimentally examined (98). Moreover, as noted above, the 130b *06635* mutant, unlike the Lp02 *lpg1993* mutant, did not exhibit hypersensitivity to lysozyme, greater resistance to NaCl, or impaired intracellular survival after entry into amoebae. Thus, the data regarding Lpg1993 and its corresponding Lp02 mutant did not negate the utility of pursuing alternative hypotheses for 06635 activity.

### Protein 06635 promotes deacetylation of the *L. pneumophila* outer surface and its lipopolysaccharide (LPS)

**Fig 4:**
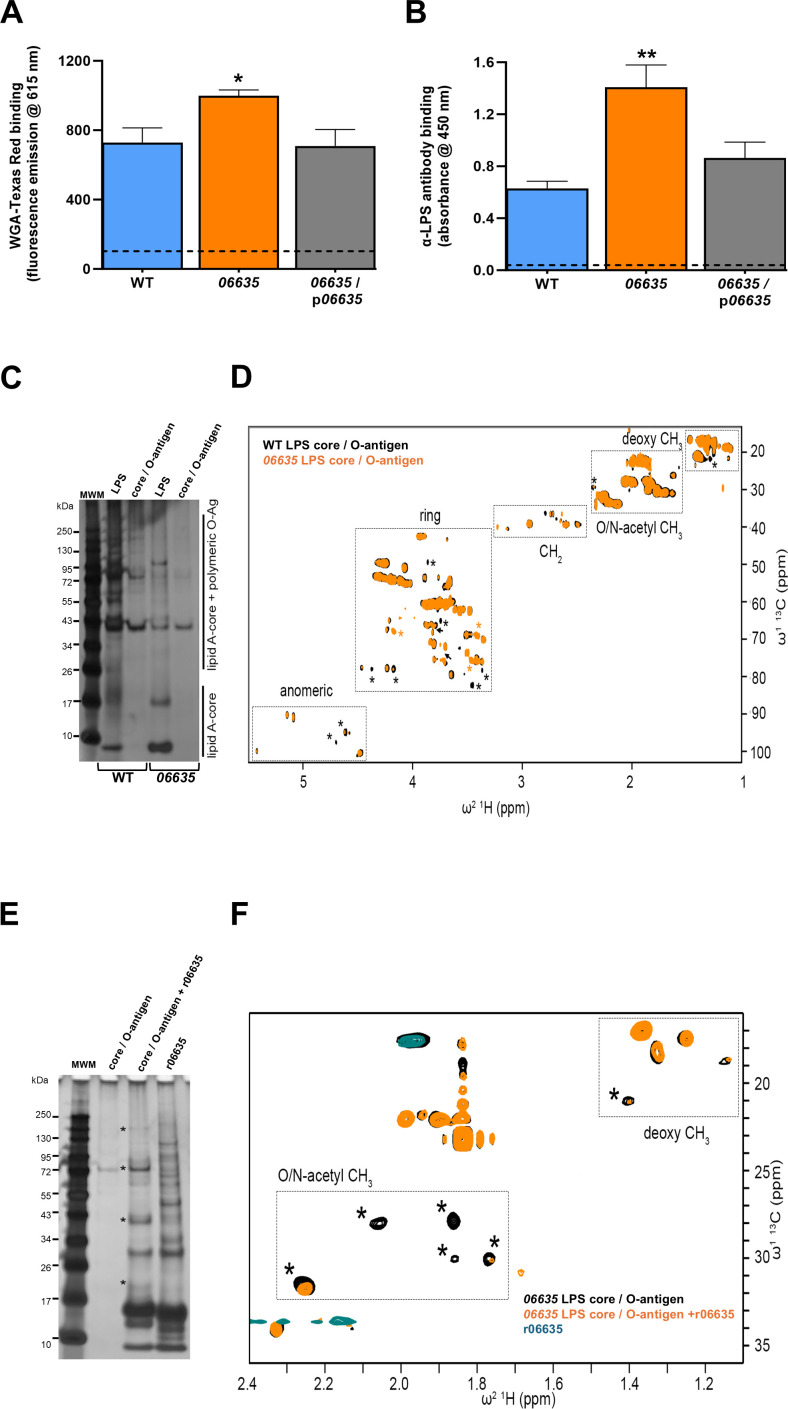
Effect of protein 06635 on *L. pneumophila* WGA binding, reactivity with MAb 3-1, and LPS. (**A**) Following growth in buffered charcoal yeast extract (BYE) broth at 37°C to early stationary phase, WT strain 130b (WT), *06635* mutant NU486 (*06635*), or NU486 containing plasmid-carried *06635* (*06635*/p*06635*) were suspended in phosphate buffered saline (PBS) with or without WGA-Texas Red. Following static incubation for 1 h at 37°C, the bacteria were washed and resuspended in fresh PBS, and the amount of bound WGA-Texas Red was quantified using a fluorimeter where excitation/emission were measured at 595/615 nm. Readings obtained from control wells containing no added bacteria are indicated by the horizontal dashed line. The data presented are the means and standard deviations from four technical replicates, and the asterisk indicates that the mutant behaved differently from the other two strains, *P* < 0.05. (**B**) Following 3 days of growth on BCYE agar at 37°C, WT 130b, the *06635* mutant, and the complemented *06635* mutant were subjected to whole-cell ELISA (four technical replicates) utilizing anti-LPS MAb 3-1. Anti-LPS-specific antibody binding was detected by secondary antibody-associated absorbance at 450 nm. Readings obtained from control wells containing no added bacteria are indicated by the horizontal dashed line. The asterisk indicates greater antibody binding for the *pdaA* mutant relative to the other strains, *P* < 0.005. (**C**) Following the growth of WT 130b (WT) and the *06635* mutant (*06635*) on BCYE agar for 3 days at 37°C, bacterial LPS was purified, and 10 µg of intact LPS or 2 µg core/O-antigen samples were examined by silver staining after SDS-PAGE. Lane 1 contains molecular weight markers (MWMs), as indicated. The bands corresponding to lipid A/core vs core/O-antigen are highlighted. (**D**) ^1^H^13^C HSQC nuclear magnetic resonance (NMR) spectra of purified core/O-antigen fragments (2 mg/mL) from WT (black) and *06635* mutant (orange) strains. Characteristic spectral range for core/O-antigen polysaccharides is highlighted. Chemical shift changes are shown as black arrows. Peak intensities that have increased (orange asterisk) or decreased (black asterisk) >50% in the mutant spectrum compared to the WT spectrum are also highlighted. (**E**) Silver-stained SDS-PA gels showing *06635* mutant core/O-antigen (0.7 mg/mL) alone, *06635* mutant core/O-antigen (0.7 mg/mL) with r06635 (r06635; 20 µM), or r06635 (20 µM) alone, after incubation at 37°C for 24 h. Lane 1 contains MWMs, as indicated. New bands that appear in the core/O-antigen sample lane when incubated with r06635, which are not present when r06635 is incubated alone, are highlighted with a black asterisk. (**F**) ^1^H^13^C HSQC NMR spectra of samples from panel **E**, highlighting the O-/N-acetyl methyl and deoxy methyl region. Peak intensities that have decreased (black asterisk) >50% in the *06635* mutant sample after treatment with r06635 are highlighted. In panels **A **through **F**, the data presented are representative of the results obtained from at least three independent experiments.

To begin to test our hypotheses for 06635 activity, we examined the *06635* mutant for altered whole-cell binding to wheat germ agglutinin (WGA), a lectin that recognizes GlcNAc (99–101). Upon incubation with WGA-Texas Red, WT strain 130b exhibited significant binding (Fig. 4A), as expected from prior tests (19). More notably, the *06635* mutant had increased WGA binding relative to WT and the complemented *06635* mutant (Fig. 4A), implying that 06635 limits GlcNAc levels on the outer surface of *L. pneumophila*.

The best known GlcNAc-containing moiety on the outer surface of *L. pneumophila* is the polysaccharide portion of LPS (102). Indeed, *N*- and *O*-acetylations occur at GlcNAc in the inner core region of the polysaccharide, but *N*- and *O*-acetylations are also present in other residues of the core (2OAc-rhamnose [2OAc-Rha]; 4-OAc-*N*-acetyl-quinovosamine [4OAc-QuiNAc]; 3OAc-GlcNAc) and within legionaminic acid (Leg) in the outer *O*-specific chain (102–111). To begin to discern if 06635 affects LPS, we performed whole-cell ELISA utilizing monoclonal antibody MAb 3-1, which recognizes acetylated forms of LPS (102, 112). WT 130b reacted with the MAb (Fig. 4B), as it had before (113). Yet, the *06635* mutant bound more MAb 3-1 when compared to WT and its complement (Fig. 4B), implying that it carries more acetylated LPS on its surface. A similar result occurred when we performed the ELISA using MAb 2-1, an anti-LPS Mab that behaves like MAb 3-1 (112) (Fig. S5A). However, when we performed the ELISA using antibodies directed against a “random” surface marker, i.e., Lcl (25), the *06635* mutant did not show elevated reactivity (Fig. S5B), indicating that it is not generally hyper-reactive in the assay. Also, when we used a *dot/icm* mutant lacking the envelope-spanning T4SS apparatus in a MAb 3-1 ELISA, it did not show increased reactivity (Fig. S5C), indicating that elevated binding to anti-LPS MAbs is not simply a byproduct of any type of altered surface.

To confirm that 06635 promotes the presence of deacetylated LPS on the *L. pneumophila* surface, we purified LPS from WT 130b and the *06635* mutant and isolated the core/O-antigen regions. Analysis of the samples at equimolar concentration by SDS-PAGE indicated that the WT sample more readily entered the gel, which was also observed for the WT core/O-antigen (Fig. 4C). The core/O-antigen samples were subjected to nuclear magnetic resonance (NMR) analysis, where ^1^H^13^C HSQC spectra showed signals in the characteristic range for core/O-antigen polysaccharides (Fig. 4D), e.g., anomeric protons (δ 4.4–5.5 ppm), sugar ring protons (δ 3.3–4.5 ppm), methylene protons (δ 2.3–3.3 ppm), O-/N-acetyl methyl protons (e.g., Leg, QuiNAc, GlcNAc; δ 1.5–2.4 ppm), and deoxy sugar methyl protons (e.g., 3-deoxy-d-manno-oct-2-ulosonic acid, 2OAc-Rha, Kdo, Leg, QuiNAc; δ 1.0–1.5 ppm) (114). However, there was a general increase in peak intensity across the WT spectrum vs the mutant spectrum, and with some new peaks appearing and others disappearing, which indicates structural changes and reduced sample aggregation (Fig. 4D). To further probe this, we cloned *06635* into *Escherichia coli* and then incubated purified recombinant protein (r06635) with the core/O-antigen isolated from the *06635* mutant. SDS-PAGE analysis showed significant increases in several band intensities in the LPS sample when incubated with r06635, which were not present for r06635 alone (Fig. 4E). Further examination by ^1^H^13^C HSQC NMR revealed clear increases of peak intensities in the *O*-/*N*-acetyl methyl region of the *06635* mutant core/O-antigen spectrum when incubated with r06635 (Fig. 4F). Thus, based on three types of assays, we conclude that 06635 promotes deacetylation of the *L. pneumophila* surface, including LPS deacetylation. Given these data and the bioinformatic analyses, we termed 06635 as PdaA, for polysaccharide deacetylase A.

### PdaA limits autoaggregation and biofilm formation by *L. pneumophila*

**Fig 5:**
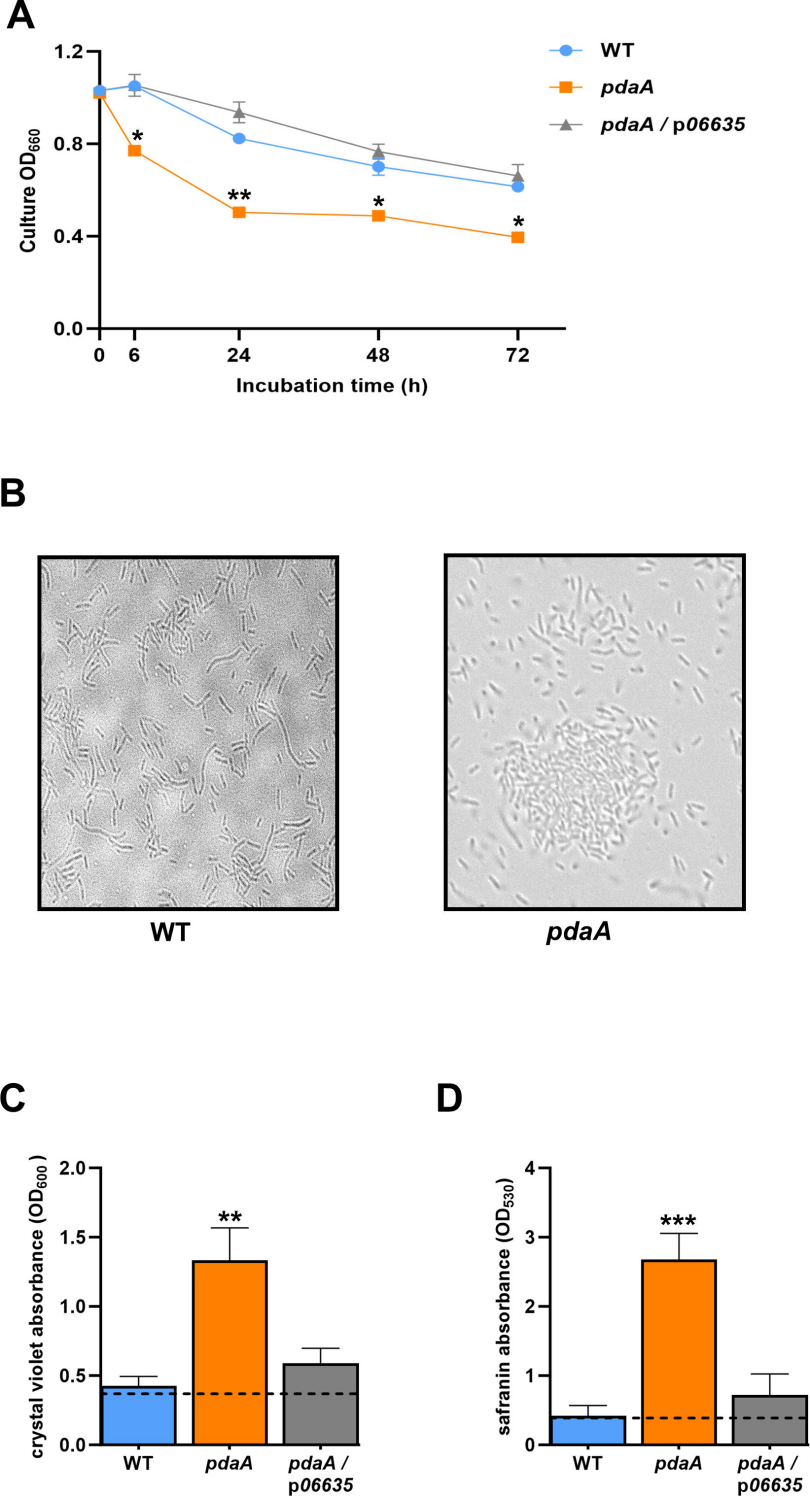
Effect of PdaA on *L. pneumophila* autoaggregation and biofilm formation. (**A**) Following 3 days of growth on buffered charcoal yeast extract (BCYE) agar at 37°C, WT strain 130b (WT), *pdaA* mutant NU486 (*pdaA*), or NU486 containing plasmid-carried *pdaA* (*pdaA*/p*pdaA*) were suspended in 10% BYE broth to an OD_660_ of ~1.0. Five-milliliter aliquots were added to glass tubes, and bacterial sedimentation at 37°C was assessed by measuring drops in the OD_660_ of the statically incubated suspensions. The data presented are the means and standard deviations from three technical replicates, and asterisks indicate that the mutant behaved differently from the other two strains at all time points after *t* = 0, *, *P* < 0.05, **, *P* < 0.005. (**B**) Following static incubation, as noted in panel **A**, strain aggregation was assessed at *t* = 48 h by microscopically examining an aliquot taken from the midpoint in the tube. (C and D) Following 3 days of growth on BCYE agar at 37°C, WT 130b, the *pdaA* mutant, and the complemented *pdaA* mutant were resuspended to an OD_660_ of ~0.2 in BYE broth, and then the suspensions were added into the wells of a 96-well polystyrene microtiter plate. After 2 days at 37°C, the amount of biofilm formed was determined by staining with crystal violet (**C**) or safranin (**D**) as read at either 600 nm or 530 nm. The readings obtained from control wells containing only medium (no added bacteria) are indicated by the horizontal dashed lines. Data presented are the means and standard deviations from six technical replicates, and asterisks indicate differences in the levels of biofilm formation between the *pdaA* mutant and the other two strains, **, *P* < 0.005; ***, *P* < 0.0005. The data in panels A–D are representative of the results obtained from at least three independent experiments.

Higher levels of acetylation in the outer core of LPS have correlated with increased surface hydrophobicity and autoaggregation (102, 103, 115, 116). Thus, strains were statically incubated in liquid, and bacterial sedimentation was monitored by a decrease in the turbidity of the cell suspension, as before (21, 117–119). Compatible with it having increased autoaggregation, the *pdaA* mutant displayed greater sedimentation (Fig. 5A). When the tubes were vortexed prior to being read in the spectrophotometer, this phenotype was lost, indicating that the mutant’s greater drop in turbidity was not due to cell lysis. Also, when the samples were viewed by microscopy, the *pdaA* mutant showed greater clumping (Fig. 5B). Next, we posited that PdaA might also impact biofilm formation, an aspect of *L. pneumophila* and others that has been linked to T2SSs and exopolysaccharides (14, 16, 38, 120–122). Using crystal violet staining (38, 122–125), the *pdaA* mutant exhibited more biofilm on plastic surfaces than WT did, and this phenotype was absent for the complemented mutant (Fig. 5C). When we measured the biofilms by staining with safranin (125, 126), a similar result was obtained (Fig. 5D). Together, these data indicated that PdaA impedes or down-regulates *L. pneumophila* autoaggregation and biofilm formation.

### PdaA promotes *L. pneumophila* resistance to serum-mediated killing and polymyxin B

**Fig 6:**
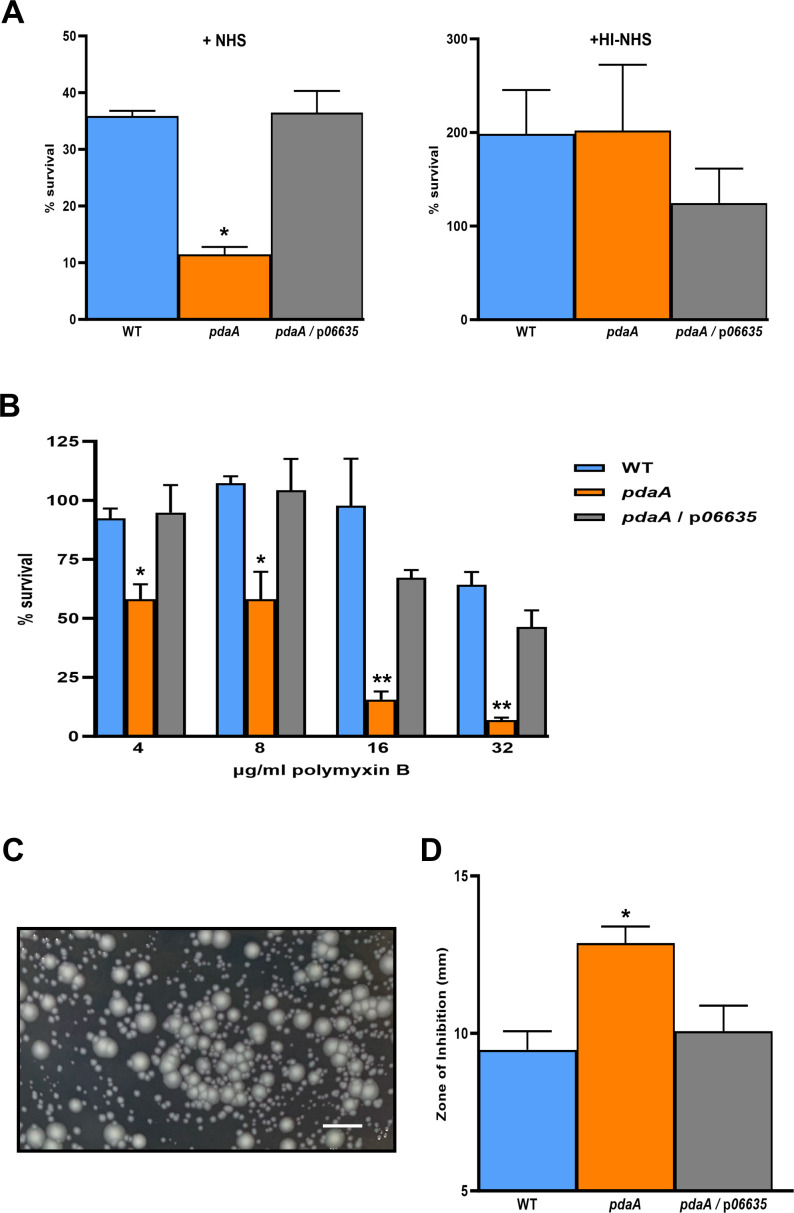
Effect of PdaA on *L. pneumophila* resistance to NHS and polymyxin B. (**A**) Following 3 days of growth on buffered charcoal yeast extract (BCYE) agar at 37°C, WT strain 130b (WT), *pdaA* mutant NU486 (*pdaA*), or NU486 containing plasmid-carried *pdaA* (*pdaA*/p*pdaA*) were suspended in 90% NHS (left) or heat-inactivated NHS (HI-NHS) (right) and then statically incubated at 37°C. After 1 day of incubation, the percentages of surviving CFU were determined by plating on BCYE agar. The data presented are the means and standard deviations from three technical replicates, and the asterisk indicates that the mutant behaved differently from the other two strains, *P* < 0.05. (**B**) Following 3 days of growth on BCYE agar at 37°C, WT 130b, the *pdaA* mutant, and the complemented *pdaA* mutant were suspended in BYE broth to an OD_660_ of 0.3 and then plated for CFU on BCYE agar vs BCYE agar containing 4, 8, 16, or 32 µg/mL of polymyxin B. After 3 days of incubation at 37°C, the percent survival of CFU on the drug-containing media vs the no-drug medium was determined. The data presented are the means and standard deviations from three technical replicates, and the asterisks indicate that the mutant behaved differently from the other two strains, with *, *P* < 0.05, and **, *P* < 0.005. (**C**) Image of *pdaA* mutant colonies on BCYE containing 32 µg/mL of polymyxin B. The scale bar corresponds to 5 mm. (**D**) Following 3 days of growth on BCYE agar at 37°C, WT 130b, the *pdaA* mutant, and the complemented *pdaA* mutant were spread onto the surface of a BCYE agar plate, and then sterile paper disks containing 5 mg/mL polymyxin B were placed onto the center of the plate. After 3 days of incubation at 37°C, the diameters of the clearing zones around the disks were measured. The data presented are the means and standard deviations from three technical replicates, and the asterisk indicates that the mutant behaved differently from the other two strains, with *P* < 0.05. The data in panels A–D are representative of the results obtained from at least three independent experiments.

Given the effect that PdaA had on the *L. pneumophila* surface, we posited that the protein might promote resistance to the killing by serum (complement), an aspect of *L. pneumophila* that is understudied (19, 33, 127–129). Although *L. pneumophila* strains are relatively resistant to serum-mediated killing (130, 131), their LPS can activate complement, and changes in the polysaccharide can trigger increased serum sensitivity (128, 129, 132, 133). Upon incubation with normal human serum (NHS), the *pdaA* mutant had reduced survival relative to WT and the complemented mutant (Fig. 6A), signifying that PdaA fosters resistance to serum. When the NHS was heat-inactivated, the differences between the strains were abolished (Fig. 6A), indicating that PdaA combats the bactericidal effects of complement. Thus, by virtue of its ability to resist killing by NHS, PdaA may be a novel *L. pneumophila* virulence factor.

For gram-negative bacteria, resistance to polymyxin-type antibiotics is often due to changes in the core region (or lipid A) of LPS, leading to more repulsion of the cationic drugs (134–140). Thus, we compared WT 130b, the *pdaA* mutant, and the complemented *pdaA* mutant for their efficiency of plating on buffered charcoal yeast extract (BCYE) agar containing increasing amounts of polymyxin B. Whether tested against 4, 8, 16, or 32 µg/mL of the drug, the *pdaA* mutant had reduced CFU relative to WT and its complement (Fig. 6B), and many of the mutant’s colonies were considerably smaller on the drug-containing agar (Fig. 6C). When we placed disks containing the antibiotic onto lawns of the bacteria and measured resultant clearing zones, the mutant again showed greater sensitivity to a range of drug concentrations (Fig. 6D). Together, these data indicated that PdaA promotes resistance to polymyxin B.

### Further phenotyping of the *lspF* T2SS mutant

To characterize the *pdaA* mutant, we had utilized a variety of assays that had not been employed before for the study of the *L. pneumophila* T2SS. Therefore, to begin to assess the overall effect of the T2SS in several of these assays, we tested the *lspF* T2SS mutant for its ability to survive in NHS, autoaggregate, and bind WGA. The *lspF* mutant was impaired relative to WT 130b for survival in NHS (Fig. S6A), a result that aligned with the behavior of the *pdaA* mutant (Fig. 6A) as well as a mutant lacking the metalloprotease ProA (33). The T2SS mutant exhibited increased autoaggregation (Fig. S6B), which is also a phenotype shown by the *pdaA* mutant (Fig. 5A). However, unlike the *pdaA* mutant (Fig. 4A), the *lspF* mutant did not have altered WGA binding (Fig. S6C), suggesting the possible existence of another T2SS substrate(s) that can counteract the effect of PdaA in some circumstances.

## DISCUSSION

The current study has demonstrated that PdaA (i) is a substrate of the *L. pneumophila* T2SS, (ii) exists in both culture supernatants and on the bacterial outer surface, (iii) is conserved within *L. pneumophila* strains and has homologs in nearly all other *Legionella* species, (iv) represents a large but heretofore uncharacterized branch of the CE-4 superfamily that includes hypothetical proteins from other pathogens, and (v) promotes deacetylation of the *L. pneumophila* outer surface. We further documented that PdaA (i) promotes infection of and replication in aquatic amoebae, (ii) impedes or down-regulates biofilm formation, which correlates with inhibiting autoaggregation, and (iii) is required for optimal resistance to polymyxin B, an antimicrobial that is present in natural environments. Thus, PdaA likely has a key role in *L. pneumophila* survival within water systems. Based on the magnitude of the *pdaA* mutant’s defect during infection of acanthamoebae, the importance of PdaA is greater than or equal to that of all previously defined T2SS substrates. From our mutant analysis, it was also clear that PdaA promotes the ability of *L. pneumophila* to resist killing by human serum and likely its complement component and thus can be classified as a virulence factor. Since the *pdaA* mutant grew normally in macrophage-like U937 cells, the *in vivo* role of PdaA may be mainly linked to bacterial survival in the extracellular spaces of the lungs. That *L. pneumophila* has an extracellular phase in the lungs (and in water habitats) is evident from many studies (5, 10, 11, 19, 141, 142), even though the *L. pneumophila* field has mostly focused its attention on intracellular infection events. Since PdaA promoted resistance to polymyxin B, we hypothesized that it might also promote *in vivo* survival of *L. pneumophila* by conferring resistance to lung cationic antimicrobial peptides. Thus, PdaA is a new T2SS substrate that impacts, in multiple important ways, the ecology and pathogenesis of *L. pneumophila*. Proteins closely related to PdaA that occur in other *Legionella* species and in other types of bacteria are likely similarly important.

For multiple reasons, we posit that the *pdaA* mutant phenotypes are due, at least partly, to the action of PdaA on LPS and, more specifically, deacetylation of the surface polysaccharide. First, multiple surface-exposed domains of *L. pneumophila* LPS are acetylated (GlcNAc, 3OAc-GlcNAc, 2OAc-Rha, 4OAc-QuiNAc, Leg), as noted earlier, and the levels of acetylated LPS (or deacetylated LPS) on the *Legionella* surface vary during extracellular and intracellular growth (102, 114, 143–146). Second, the *pdaA* mutant had increased binding to MAbs that recognize acetylated forms of LPS. Third, the *pdaA* mutant had increased sensitivity to polymyxin B, and as noted above, polymyxin resistance has been linked to changes in the charge of surface polysaccharide. Fourth, based on SDS-PAGE and NMR analyses, *pdaA* mutant LPS had changes in structure and increased aggregation. Finally, based on studies of *L. pneumophila* and various others, LPS broadly influences bacterial growth in hosts, biofilm formation, and NHS resistance, as noted above. To our knowledge, no secreted enzyme of *L. pneumophila* nor any CE-4 deacetylase has been linked before to changes in LPS or to changes at the cognate bacterial surface (103, 110, 147, 148), although unrelated polysaccharide deacetylases on phage spikes act on LPS as a step toward phage attachment (149, 150). However, a role for a T2SS substrate in modulating LPS structure was recently reported, when it was found that TssM of *Burkholderia* species can reverse LPS ubiquitylation (14, 151). Thus, combining this study with our work, the conceptual framework for examining the modes of action of secreted enzymes is expanded. Theoretically, having PdaA act on LPS that is already assembled on the surface would represent a more rapid and perhaps localized mechanism for adjusting to environmental changes as opposed to making *de novo* different forms of LPS.

Although more work is needed to test if the various mutant phenotypes listed above are due to diminished LPS deacetylation, our results lead to a working model in which PdaA helps *L. pneumophila* alternate between its major states of environmental existence. We propose that when PdaA is secreted by the T2SS, the protein (whether tethered to the bacterial surface or in nearby extracellular milieu) deacetylates surface moieties, resulting in a bacterial form that does not autoaggregate and favors growth in amoebae. However, when PdaA is repressed, the resultant reduction in surface deacetylation leads to a different bacterial form that is conducive to biofilm formation. Within this framework, we posit that the level of *pdaA* expression is responsive to changes in environmental stimuli. Microarray analysis of a *L. pneumophila rpoS* mutant revealed a sevenfold increase in expression of the gene (152), suggesting a role for PdaA in responding to acid, oxidative stress, high osmolarity, or nutrient limitation. The fact that PdaA promotes resistance to NHS not only means that the protein is a new virulence factor but provides a striking example of how the evolution of structures and processes in response to environmental pressures can increase the virulence of an “accidental” pathogen such as *L. pneumophila*.

As noted earlier, after we were far advanced in our study of PdaA, it was reported that the protein’s equivalent from *L. pneumophila* strain Lp02 (i.e., Lpg1993) acts as a PG deacetylase, which can influence the functioning of the Dot/Icm T4SS (98). However, the extracellular secretion of Lpg1993 by Lp02 was not previously examined (98). Moreover, as was documented above, the 130b *pdaA* mutant did not exhibit hypersensitivity to lysozyme nor show evidence of having an impaired Dot/Icm T4SS. Although it is possible that the differences in results obtained here vs the Lp02 *lpg1993* mutant study could be due to variations in the preparation of the bacteria prior to testing and/or other methodological aspects, it should also be noted that, unlike the 130b strain used in our study, Lp02 is not a WT strain but rather a laboratory-derived variant of Philadelphia-1 that has sustained a 45 kb deletion, encompassing the Lvh T4SS locus (5, 153) and other genes, and other mutations that affect *rpsL, ndh, leuS,* and *nuoG* (55, 154, 155). Indeed, among other things, strain Lp02 grows much more poorly on low-iron media and exhibits less virulence when compared to its WT parent Philadelphia-1 and/or strain 130b (26, 156, 157). Thus, we do not believe that the data regarding Lpg1993 negate the results and implications of our study. For example, PdaA and its homologs may localize or act differently in different strains, e.g., in WT vs a lab derivative such as Lp02. Alternately, the proteins may target multiple substrates, e.g., PG when in the periplasm (on its way to secretion out of the cell) vs LPS and environmental substrates when surface-associated or fully extracellular. Indeed, it is tantalizing to ponder the novel possibility that some T2SS substrates may have one role in the periplasm and another role after passage into extracellular spaces. Thus, future work should strive to build on the results of both the current and prior studies, e.g., assaying PdaA for activity against a wider range of substrates.

Besides helping define PdaA, the current proteomic analysis expanded our knowledge regarding the size and potential functions of the *L. pneumophila* T2SS. Besides confirming the T2SS dependency of 29 proteins that had been previously seen in *L. pneumophila* supernatants, we detected 65 additional proteins whose presence in supernatants was diminished by the absence of the T2SS. Although some of these 65 (Table 2) may not be true T2SS substrates but rather proteins whose presence in supernatants is only indirectly influenced by the T2SS, the documented output of the *L. pneumophila* T2SS now stands at approximately 120, which is among the largest known (14, 15, 158, 159). The expanded T2SS output is predicted to include an interesting array of proteins. Some, such as the highly expressed putative proteases/peptidases encoded by *00985* and *04070*, are like previously described substrates. Yet, others may confer new activities, e.g., the putative cholylglycine hydrolase/penicillin V acylase (160) from *12245*, diphosphohydrolase from *11405* (161), 5-nucleotidase from *02545*, glutaminase from *08370*, and kinase from *07015*. Moreover, there are new novel proteins that lack similarity to any known proteins, e.g., the highly expressed 05010 and 03825. In addition to uncovering these many new T2SS substrates, the current study has, through its characterization of PdaA, documented the first linkage between a T2SS substrate and bacterial resistance to polymyxins. Finally, regarding the PdaA paralog 13095, it will be interesting for future work to discern the degree to which the function of secreted 13095 overlaps with or counteracts that of secreted PdaA.

## MATERIALS AND METHODS

### *L. pneumophila* strains, media, and extracellular growth

WT strain 130b (American Type Culture Collection [ATCC] strain BAA-74) was previously described, as were its *proA* mutant AA200, *lspF* mutant NU275, and *dotA* mutant NU428 (27, 28, 162). These strains and all new mutants of 130b (below) were grown at 37°C on BCYE agar or in BYE broth (163). The extracellular growth of strains was determined by monitoring the OD_660_ of BYE cultures (125).

### Mutant constructions and genetic complementation

Mutants that have deletions of *06635* (NU486), *11870* (NU487), *13770* (NU488), *08770* (NU489), *00420* (NU490), *12705* (NU491), or *06500* (NU492) were made by allelic exchange utilizing overlap extension PCR (OE-PCR), as before (28, 125, 164). First, ~1 kb fragments of the 5′ and 3′ regions flanking each of the target open reading frames (ORFs) were PCR-amplified from 130b DNA using HiFi Taq polymerase (Life Technologies) and primers CA1 and CA2 for 5′ *06635*, CA3 and CA4 for 3′ *06635*, CA7 and CA8 for 5′ *11870*, CA9 and CA10 for 3′ *11870*, CA13 and CA14 for 5′ *13770*, CA15 and CA16 for 3′ *13770*, CA19 and CA20 for 5′ *08770*, CA21 and CA22 for 3′ *08770*, CA25 and CA26 for 5′ *00420*, CA27 and CA28 for 3′ *00420*, CA31 and CA32 for 5′ *12705*, CA33 and CA34 for 3′ *12705*, CA37 and CA38 for 5′ *06500*, and CA39 and CA40 for 3′ *06500*. The sequences for primers listed above and below are in Table S5A. Second, a kanamycin (Kn) resistance cassette was PCR-amplified from pKD4 using either primers CA5 and CA6 for use in the eventual mutation of *06635*, primers CA11 and CA12 for mutating *11870*, CA17 and CA18 for *13770,* CA23 and CA24 for *08770*, CA29 and CA30 for *00420*, CA35 and CA36 for *12705*, or CA41 and CA42 for *06500*. Next, we did OE-PCR to combine the 5′ and 3′ regions of each ORF with its Kn-resistance cassette. PCR products were gel-purified and ligated into pGEM-T Easy (Promega) for mutation of *11870, 13770, 08770, 00420*, and *12705* or retained as a linear fragment for mutating *06635* and *06500*. In all cases, 10 µg of DNA was used for transformation of WT 130b. Bacteria likely containing an inactivated gene were obtained by plating the transformation mixtures on BCYE agar containing Kn. Mutation verification was done by multiple PCRs using either primers specific for each of the targets (above) or those primers in conjunction with CA43 and CA44, which mark the Kn-resistance cassette. A mutant deleted for *proA* and *lspF* (NU493) was made in a similar way. Approximately 800 bp fragments of the 5′ and 3′ regions flanking *lspF* were PCR-amplified using CA45 and CA46 for 5′ *lspF* and CA47 and CA48 for 3′ *lspF*. A gentamicin (Gm)-resistance cassette was amplified from pR6Kgent using CA49 and CA50. OE-PCR combined the 5′ and 3′ regions of *lspF* with the resistance cassette. Linear DNA containing the mutated allele was used for transformation of AA200. Bacteria putatively containing inactivated *lspF* were recovered on BCYE agar with Gm. Verification of altered *lspF* was done by PCR using the primer pairs CA45 and OR78 and CA48 and OR77.

Complementation of the *06635* mutant was done as before (44, 125). A 1,077 bp fragment containing the *06635* ORF with its native promoter region (but no other intact gene or sRNA) was PCR-amplified using CA51 and CA52. The *06635-*containing DNA was then cloned into pMMBGent, yielding p*06635*. PCR using the vector-specific primers OR77 and OR78 and DNA sequencing confirmed p*06635’s* construction. Plasmid p*06635* was electroporated into NU486, and transformants containing p*06635* were obtained by plating on BCYE agar containing Gm.

### Intracellular infection assays

*A. castellanii* (ATCC 30234) and *V. vermiformis* (ATCC 50237) were maintained as before (40, 45). *L. pneumophila* growth within amoebal co-cultures was monitored as previously described (40, 125). Determinations of bacterial entry into amoebae and early intracellular survival were also done as before (45, 164). Finally, amoebae were infected with green fluorescent protein (GFP)-expressing legionellae (in media containing 1 mM isopropyl-beta-d-thiogalactopyranoside [IPTG] for GFP induction), and increases in intracellular fluorescence were monitored, as before (45, 125, 164). To that end, a GFP-expressing plasmid was introduced into WT and *06635* mutant by electroporation (28, 164). U937 cells (ATCC CRL-1593.2) were converted to a macrophage-like state by treatment with phorbol myristate acetate, and then infected with *L. pneumophila* as before (28, 125).

### Immunoblot analysis

To track 06635 secretion, a FLAG tag was added to the C-terminus of the protein. Plasmids encoding intact *06635* with an added 3′-FLAG were made by either using primers CA53 and CA54 to PCR-amplify a tagged gene from 130b DNA for cloning into Gm-resistant pMMBGent, yielding p06635-FLAG1, or utilizing CA55 and CA56 to PCR-amplify the gene for cloning into chloramphenicol (Cm)-resistant pMMB2002 (26), yielding p06635-FLAG2. PCR using vector-specific primers OR77 and OR78 and plasmid sequencing (Plasmidsaurus) were done to confirm the plasmid constructions. p06635-FLAG1 and p06635-FLAG2 were electroporated into WT 130b, *proA* mutant AA200, and *lspF* mutant NU275, and transformants carrying a plasmid were recovered on BCYE agar containing Gm or Cm. p06635-FLAG2 was similarly introduced into *proA lspF* mutant NU493. Strains encoding 06635-FLAG were inoculated to an OD_660_ of ~0.3 in 50 mL of BYE broth containing 1 mM IPTG (to induce 06635-FLAG expression) and incubated at 37°C with shaking for 14 h. As before (26), supernatant proteins were collected by centrifugation, sterilized by passage through 0.22 µm filters (EMD Millipore), concentrated 20-fold by isopropanol precipitation, and suspended in Laemmli buffer. Cell pellets from the centrifugation of the cultures were also suspended in Laemmli buffer. Supernatant and cell pellet samples (corresponding to equal amounts of culture per strain) were subjected to 15% SDS-PAGE and then immunoblotted with a 1:20,000 dilution of rabbit anti-FLAG antibody (Invitrogen cat. #740001) in 1% skim milk–Tris-buffered saline with Tween-20 (TBST). Secondary IRDye 680 goat anti-rabbit IgG antibody (LI-COR Biosciences) was used at a 1:20,000 dilution in milk-TBST. Blots were imaged using the LI-COR Biosciences Odyssey Fc Imaging System.

### Proteomic analysis

WT 130b and *lspF* mutant were grown in BYE broth to mid-log (OD_660_ ~0.7). Supernatants were filter sterilized and, after the addition of protease inhibitors (Sigma #P8465), analyzed by label-free quantification (LFQ) proteomics. Samples were first subjected to in-solution digestion and then treatment with cold acetone. Protein pellets were suspended in 100 µL of 8 M urea/0.4 M ammonium bicarbonate (AmBic), and after the addition of 4 µL of 100 mM dithiothreitol, samples were incubated at 50°C for 45 min. After the addition of 6 µL of indole-3-acetic acid, samples were placed at 25°C in the dark for 45 min. Next, 290 µL of AmBic mass spectrometry (MS)-grade trypsin (Promega) was added at an enzyme-to-substrate ratio of 1:50 for overnight digestion at 37°C. Samples were then dried and de-salted using C18 spin columns. Peptides were analyzed using a Vanquish Neo nano-LC coupled to an Orbitrap Exploris 240 hybrid quadrupole-orbitrap mass spectrometer (Thermo Fisher). Separation was done using a UHPLC C18 column (Ion Opticks, AUR3-15075C18-CSI). For each run, 1 µg of peptide sample was injected. Electrospray ionization was done using a Nanospray Flex Ion Source (Thermo Fisher, ES071) at a positive static spray voltage of 2.2 kV. Peptides were eluted at a flow rate of 200 nL/min using an increasing organic gradient to separate peptides based on hydrophobicity. Buffer A was 0.1% formic acid in Optima liquid chromatography-mass spectrometry (LC-MS)-grade water, and buffer B was 80% acetonitrile, 19.9% Optima LC-MS-grade water, and 0.1% formic acid. Method duration was 120 min. The mass spectrometer was controlled using Xcalibur and operated in a positive polarity. Full scan (MS1) settings used mass range 350–1,600 *m/z*, radio frequency (RF) lens 60%, orbitrap resolution 120,000, normalized automatic gain control (AGC) target 300%, maximum injection time of 25 ms, and a 5E3 intensity threshold. Data-dependent acquisition by TopN was done by higher-energy collisional dissociation of isolated precursor ions with charges of 2^+^ to 5^+^ inclusive. MS2 settings were dynamic exclusion mode duration 30 s, mass tolerance 5 ppm (both low and high), 2 s cycle time, isolation window 1.5 *m/z*, 30% normalized collision energy, orbitrap resolution 15,000, normalized AGC target 100%, and maximum injection time of 50 ms. Proteins were identified from the MS raw files using the Mascot search engine (Matrix Sci., v.2.5.1). MS/MS spectra were searched against the SwissProt *L. pneumophila* database. All searches included carbamidomethyl cysteine as a fixed modification and oxidized methionine, deamidated asparagine and aspartic acid, and acetylated N-term as variable modifications. Two missed tryptic cleavages were allowed. A 1% false-discovery rate cutoff was applied at the peptide level. Only proteins with a minimum of two peptides above the cutoff were considered further. Identified peptides/proteins were visualized by Scaffold software (v.5.0, Proteome Software). Samples were acquired on MS with duplicate injections, and data were searched against a specific database using the MaxQuant application. LFQ was obtained by LFQ MS1 intensity. Results were filtered with a minimum of two unique peptides. Data from three biological replicates were averaged and transformed to log2 and then grouped and filtered for proteins with two values in at least one group. For statistics, the *t*-test was applied using *P* < 0.05 and fold change (FC) > 2 to discern which proteins were significantly up- or downregulated. To depict the distribution of the protein differences, the data were prepared as a volcano plot.

### Whole-cell ELISA

Immunodetection of proteins on the surface of *L. pneumophila* was done as before (19, 25). Primary antibodies were used at the following dilutions: anti-FLAG at 1:500, MAb 3-1 at 1:1,000, MAb 2-1 at 1:40,000, and anti-Lcl at 1:1,000 (25, 112, 165). Secondary anti-rabbit and anti-mouse antibodies (Cell Signaling #7074S and 7076S) were used at 1:1,000.

### Assays for bacterial sensitivity to NaCl and lysozyme

The sensitivity of *L. pneumophila* to NaCl was assessed similarly to past studies (166–169). Bacteria were grown with shaking in 20 mL of BYE broth at 37°C for ~18 h to an OD_660_ of ~3.5. After the cultures were normalized to an OD_660_ = 0.3 by adding BYE broth, they were subjected to a 10-fold serial dilution in phosphate buffered saline (PBS), and 100 µL aliquots were plated on BCYE agar with or without 0.65% NaCl. Later, the ratio of CFU on the salt-containing BCYE agar over the CFU on standard agar was ascertained. To judge sensitivity to lysozyme, legionellae were grown in 20 mL of BYE broth at 37°C to an OD_660_ of ~3.8. After the cultures were brought to an OD_660_ = 0.3, they were centrifuged, and the resultant pellets were resuspended in 25 mM Tris-HCl (pH 8.0), giving a concentration of ~4 × 10^5^ CFU/mL. Then, 0.5 mL of each suspension was combined with 0.5 mL of 25 mM Tris-HCl (pH 8.0) containing varying amounts of chicken egg white lysozyme (Sigma # L6876). After 30 min of static incubation at 37°C, the percentage of surviving bacteria (relative to the inoculum) was determined by plating for CFU on BCYE agar. As in prior studies (170), Tris-HCl permeabilizes the bacterial outer membrane, allowing lysozyme to access PG in the periplasm.

### Assay for WGA binding

*L. pneumophila* binding to WGA was determined analogously to prior studies, with modifications (69). Bacteria were grown with shaking in 10 mL of BYE broth (in a 125 mL flask) at 37°C for ~15 h to an OD_660_ of ~3.0. Each culture was transferred to a 15 mL conical tube and centrifuged at 5,000 × *g* for 5 min. Cell pellets were resuspended in ~10 mL of PBS to an OD_660_ = 0.7. Fifty microliters of each suspension was combined with 24 µL of a 1 mg/mL stock of Texas-Red WGA (Thermo Fisher) and 26 µL of PBS in a 200 µL polypropylene tube, and the mixtures were statically incubated at 37°C for 1 h. After another centrifugation, the bacteria were washed twice with 100 µL of PBS and then resuspended in 100 µL of PBS. Finally, the cell suspensions were transferred into the wells of a 96-well black, flat-bottom polystyrene microtiter plate (Costar), and the fluorescence of bound Alexa Fluor 488-WGA was read at 615 nm.

### Purification of LPS and its incubation with r06635

*L. pneumophila* LPS was purified as before, with minor modifications (114). After growth on BCYE agar for 3 days at 37°C, bacteria were scraped from the plates, washed in PBS, followed by H_2_O, and then lyophilized. Cells were resuspended in 10 mL of 50 mM sodium phosphate (pH 7.0), 5 mM MgCl_2_, with 0.6 mg/mL lysozyme at 4°C for 16 h. This was followed by the addition of DNase I (10 U, Thermo Fisher) and RNase (10 U, Sigma) and incubation for 2 h at 37°C. Next, proteinase K (10 U, NEB) was added, and the incubation continued overnight at 37°C. Samples were heated at 95°C for 10 min and, after the addition of an equivalent volume of 90% (vol/vol) phenol, incubated at 68°C with vigorous shaking for 15 min. Samples were cooled on ice, centrifuged at 8,500 × *g* for 15 min at 25°C, and aqueous phase retained. H_2_O was added to the organic phase, and the extraction was repeated twice. After extensive dialysis in H_2_O for 2 days, samples were lyophilized, resuspended in 0.5 mL 0.375 M MgCl_2_ in 95% (vol/vol) ethanol, and stored at −20°C for 4 h. After centrifugation at 12,000 × *g* for 15 min at 4°C, the purified LPS pellet was resuspended in H_2_O and lyophilized. To hydrolyze lipid A from the core/O-antigen, samples were resuspended in 40 mM ammonium acetate (pH 4.4) and incubated at 100°C for 4 h. After centrifugation at 12,000 × *g* for 15 min at 25°C, supernatants having purified core/O-antigen were retained. To remove residual ammonium acetate, two more lyophilizations and resuspensions in D_2_O were done.

Gene *06635*, minus the region encoding the signal sequence, was synthesized by GenScript (Table S5B) and cloned into the C-terminally His_6_-tagged pET28b vector (Novagen) using *NcoI* and *XhoI* sites. Protein expression was carried out in *E. coli* BL21 (DE3) (NEB). Cells were grown in the presence of 50 µg/mL Kn at 37°C in Luria-Bertani (LB) broth, and expression was induced with 0.5 mM IPTG at an OD_600_ of 0.6. Cells harvested after growth overnight at 18°C were resuspended in 20 mM Tris-HCl pH 8, 200 mM NaCl, 5 mM MgCl_2_, 1 mg/mL DNase I, and 5 mg/mL lysozyme and lysed by sonication. Protein was purified using nickel affinity chromatography (Qiagen) and dialyzed into PBS. The *06635* mutant core/O-antigen LPS sample (2 mg/mL) in 100% D_2_O was diluted threefold with protein in PBS to a final 06635 concentration of 20 µM and incubated while shaking at 37°C for 24 h. As controls, the core/O-antigen sample was diluted in PBS without 06635, and 100% D_2_O without core/O-antigen was diluted with 06635 in PBS. Samples were boiled for 10 min to precipitate r06635 and then centrifuged at 12,000 × *g* for 15 min at 4°C. Supernatants were retained for NMR and SDS-PAGE analyses.

### NMR spectroscopy and silver-stained SDS-PAGE

^1^H^13^C HSQC NMR experiments were done at 25°C on either a Bruker Avance III HD 800 MHz spectrometer (for core/O-antigen samples) or a Bruker Avance III HD 700 MHz spectrometer (for core/O-antigen and r06635), equipped with cryoprobes. Spectra were obtained using TOPSPIN and analyzed using CCPNMRv3 (171). Detection of LPS species by silver staining after SDS-PAGE was done as before (115).

### Biofilm and autoaggregation assays

*L. pneumophila* biofilm formation on plastic surfaces within microtiter plates was determined using crystal violet and safranin staining, as before (26, 125). *L. pneumophila* autoaggregation was also assessed as before (26).

### Assays for bacterial sensitivity to NHS and polymyxin

*L. pneumophila* sensitivity to NHS was determined as before (128, 129). Strains were resuspended in PBS to an OD_660_ = 0.3, which corresponds to ~1 × 10^9^ CFU/mL. After a 1,000-fold dilution in PBS, 300 µL of each cell suspension was added to 2.7 mL of NHS (Sigma # s1-M) in a 15 mL conical tube, and the mixtures were statically incubated at 37°C. After 24 h, remaining CFUs were enumerated by plating aliquots onto BCYE agar, and the ratio of CFU after NHS treatment over CFU prior to NHS treatment was determined. To test the effect of heat-inactivated NHS, the NHS was incubated at 55°C for 15 min and then used as above. *L. pneumophila* sensitivity to polymyxin B was assessed using modifications to past studies (172, 173). Bacteria suspended in BYE broth were plated for CFU on BCYE agar containing varying amounts of polymyxin B (US Biological P4400-01), and after 3 days of incubation at 37°C, the ratio of CFU on the drug-containing media over CFU on the standard medium was determined, and the sizes of colonies were imaged. Also, bacteria were spread onto BCYE agar, and then paper disks (Becton Dickinson) that had been soaked with two 10 µL aliquots of 5 mg/mL polymyxin B were placed onto the agar. After 3 days of incubation at 37°C, the diameters of the clearing zones across the disks were measured.

### *In silico* analyses

Predictions regarding protein secretion were obtained using SignalP 6.0 (23, 174). BLASTP at NCBI was used to identify homologs of 06635 in *L. pneumophila* strains, other *Legionella* species, and the general genome database. 06635-related proteins in species of *Berkiellales*, *Coxiellales*, *Diplorickettsiales*, *DSM-16500*, *Francisellales, Legionellales*, and *Piscirickettsiales* were identified and organized on a phylogenetic tree, as before (26). A predicted structure for 06635 was made using AlphaFold 3 (85) and rendered with ChimeraX software (v.1.9) (175). To discern possible relationships to known structures, we submitted the predicted structure to the DALI server (v.5) (176), as before (26, 80, 164).

### Statistical methods

Experiments utilized ≥3 technical replicates, and the values obtained were given as the means and standard deviations. Unless indicated differently above, *P*-values were determined by Student’s *t*-tests.

## SUPPLEMENTAL MATERIAL

Supplemental material mbio.01393-25-s0001.pdf Fig. S1-S6; Tables S1, S2, S4, and S5.

Table S3 mbio.01393-25-s0002.xlsx Full set of supernatant proteins detected in the current proteomic analysis.

ASM does not own the copyrights to Supplemental Material that may be linked to, or accessed through, an article. The authors have granted ASM a non-exclusive, world-wide license to publish the Supplemental Material files. Please contact the corresponding author directly for reuse.
