# Erratum for Adams et al., “*Legionella pneumophila* type II secretome reveals a polysaccharide deacetylase that impacts intracellular infection, biofilm formation, and resistance to polymyxin- and serum-mediated killing”

**DOI:** 10.1128/mbio.02034-25

**Published:** 2025-07-23

**Authors:** Carlton O. Adams, Jackson A. Campbell, Beichang Zhang, Leanne Cleaver, Sarah B. Bier, Joshua Mayoral, Richard C. White, James A. Garnett, Nicholas P. Cianciotto

## ERRATUM

Vol. 16, no. 7, e01393-25, 2025, https://doi.org/10.1128/mbio.01393-25. Page 21, “Proteomic analysis,” sentence 1: “mid-log (OD_660_ ~0.7)” should read “early-stationary (OD_660_ ~3.2).”

