# Supplementary material for: Legionella pneumophila type II secretome reveals a polysaccharide deacetylase that impacts intracellular infection, biofilm formation, and resistance to polymyxin- and serum-mediated killing
Source: mBio. 2025 Jun 20;16(7):e01393-25. doi: 10.1128/mbio.01393-25 (PMC12239567; doi:10.1128/mbio.01393-25)
Supplement: Supplemental material — Fig. S1-S6; Tables S1, S2, S4, and S5. [file mbio.01393-25-s0001.pdf]

A

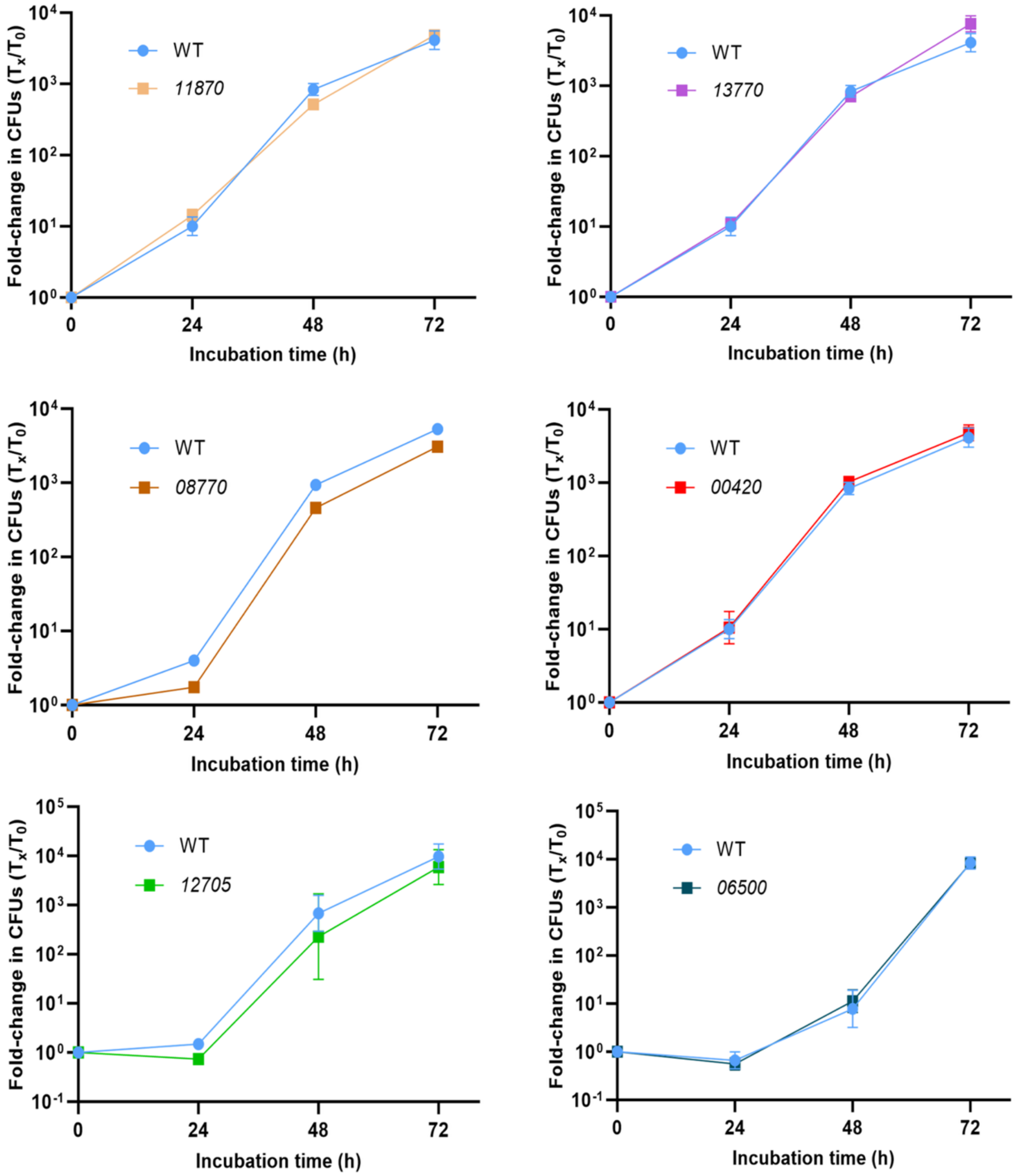

WT
11870
13770
08770
00420
12705
06500
Fold-change in CFUs (T_x/T_0)
Incubation time (h)
0
24
48
72
10^{-1}
10^0
10^1
10^2
10^3
10^4
10^5

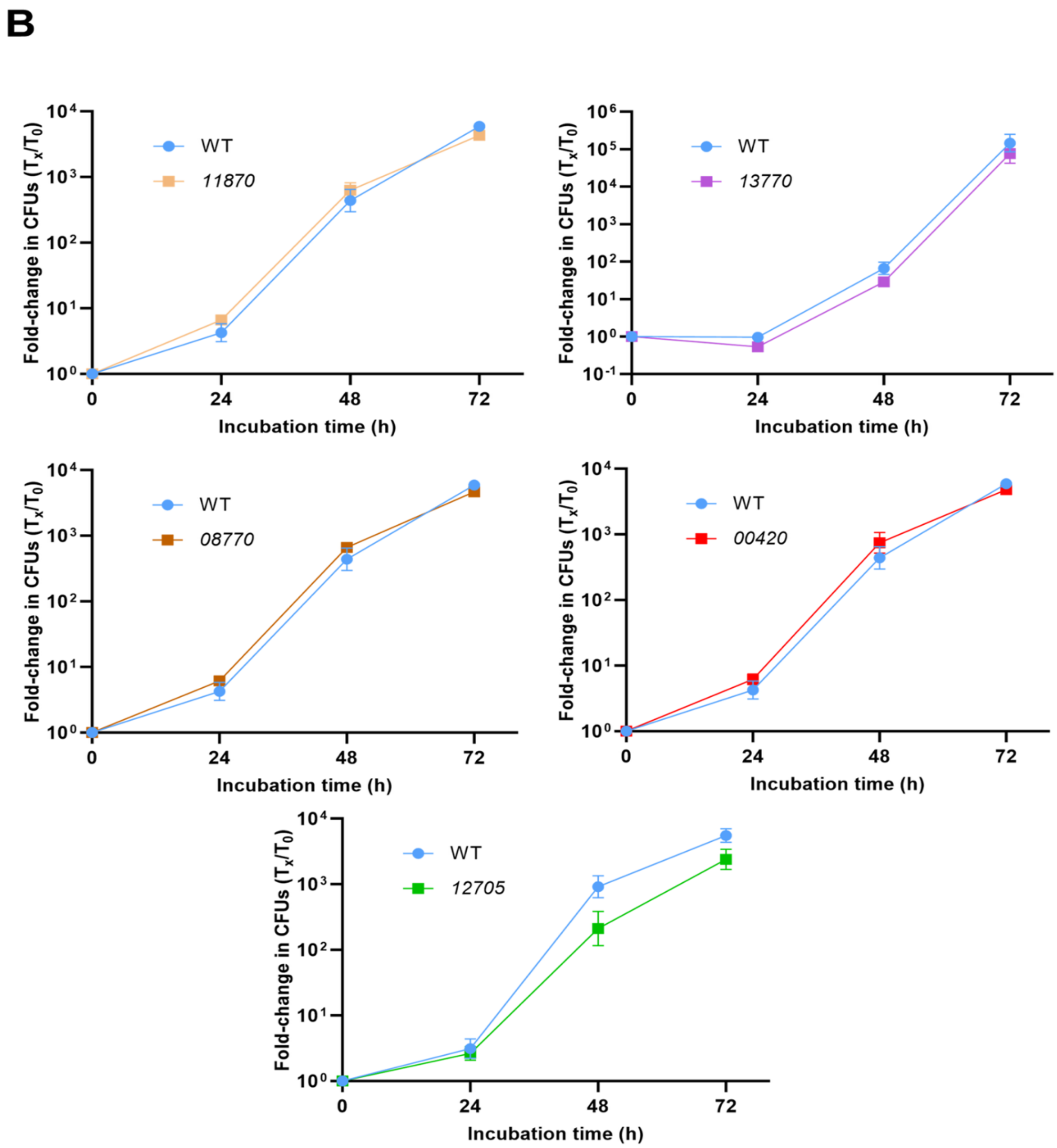

FIG S1. Effect of *11870, 13770, 08770, 00420, 12705,* and *06500* on *L. pneumophila* infection of amoebae. (A - B) Monolayers of *A. castellanii* (A) or *V. vermiformis* (B) were infected with WT strain 130b (WT), *11870* mutant NU487 (*11870*), *13770* mutant NU488 (*13770*), *08770* mutant NU489 (*08770*), *00420* mutant NU490 (*00420*), *12705* mutant NU491 (*12705*), or *06500* mutant NU492 (*06500*) at a MOI = 0.1, and then immediately (i.e., $t = 0$) and at 24, 48, and 72 h post-inoculation, aliquots taken from the culture supernatants were assessed for bacterial numbers by plating for CFU on BCYE agar. Because *L. pneumophila* does not replicate in the medium, increases in CFU are due to bacterial growth in the amoebae. The values presented in each panel are the means and standard deviations obtained from three technical replicates. The data presented are representative of the results obtained from at least two independent infection experiments.

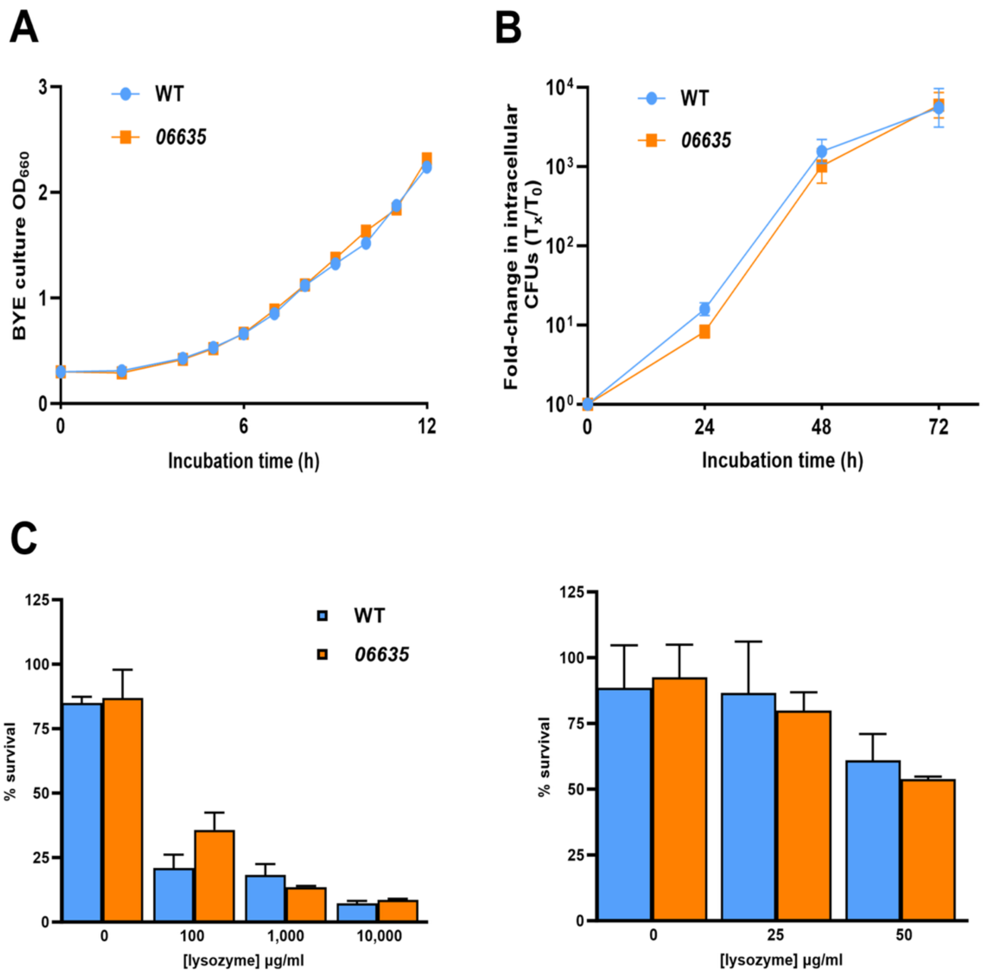

FIG S2. Effect of *06635* on *L. pneumophila* growth in BYE broth, infection of human macrophages, and sensitivity to lysozyme. (A) Following 3 d of growth on BCYE agar at 37°C, WT strain 130b (WT) and the *06635* mutant NU486 (*06635*) were inoculated into BYE broth and then bacterial growth was monitored spectrophotometrically. (B) Monolayers of PMA-differentiated U937 cells were infected with WT 130b or the *06635* mutant at a MOI = 0.5. After a 2-h incubation to allow for bacterial entry, the infected wells were washed to remove remaining extracellular bacteria, and then at that *t* = 0, 24, 48, and 72 h post-inoculation, the macrophages were lysed and bacterial numbers in the wells determined by plating for CFU on BCYE agar. Since *L. pneumophila* does not grow in the assay medium, the increases in CFU are the result of growth in the macrophages. (C) Following growth to late stationary phase in BYE broth at 37°C, WT 130b and the *06635* mutant were resuspended in Tris-buffer containing either 0, 100, 1,000, or 10,000 µg/ml of lysozyme (left panel) or 0, 25, or 50 µg/ml of lysozyme (right panel). Following 30 min of static incubation at 37°C, the percentage of surviving bacteria (relative to the starting inoculum) was determined by plating for CFU on BCYE agar. In (A) – (C), values presented are the means and standard deviations obtained from three technical replicates. The data presented are representative of the results obtained from two (A) or at least three (B – C) independent experiments.

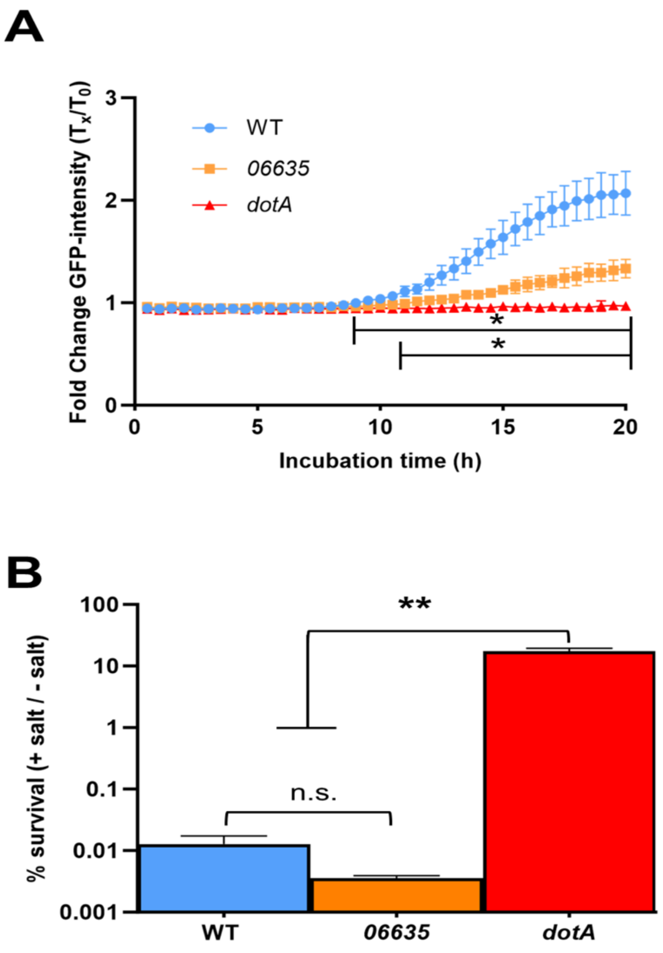

FIG S3. Comparison of the effects of *06635* and *dotA/icm* T4SS mutations on *L. pneumophila* growth within amoebae and sensitivity to NaCl. (A) Monolayers of *A. castellanii* were infected with GFP-expressing WT, *06635* mutant NU486, or *dotA* mutant NU428 at an MOI = 20. After centrifugation and a 1-h incubation to permit *L. pneumophila* uptake, gentamicin was added to kill any remaining extracellular bacteria. Fluorescence emanating from the intracellular legionellae was monitored every 30 min for the next 20 h, and the fluorescence values obtained were normalized to the GFP signal at $t = 0$ following gentamicin treatment. The values presented are the means and standard deviations from six technical replicates. The asterisk above the upper horizontal line denotes the significant difference emerging between the mutants vs WT, whereas the asterisk above the lower horizontal line signifies when the *06635* mutant is different from the *dotA* mutant; $P < 0.05$. (B) Following growth in BYE broth to stationary phase, WT 130b, the *00635* mutant, and the *dotA* mutant were plated for CFU on BCYE agar vs BCYE agar containing 0.65% NaCl. After 3 d of incubation at 37°C, the percent survival of CFU on the salt-containing medium vs. the no-salt medium was determined. The data presented are the means and standard deviations from three technical replicates, and the asterisks indicate that the *dotA* mutant behaved differently from the other two strains, with $P < 0.005$. The data presented in (A) and (B) are representative of the results obtained from two independent experiments.

A

Protein	Species	E value	% Similarity	% Identity	Accession
Peptidoglycan deacetylase	*Burkholderia pseudomallei*	4E-95	62	43	ALJ70551.1
Peptidoglycan deacetylase	*Helicobacter pylori*	2E-21	43	25	GLP31361.1

B

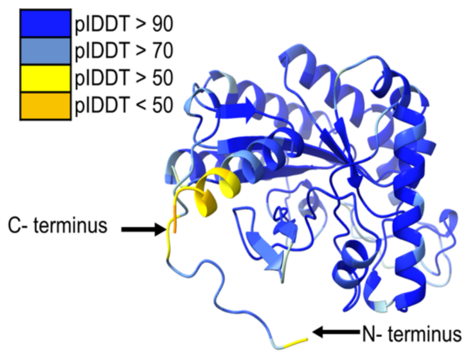

C

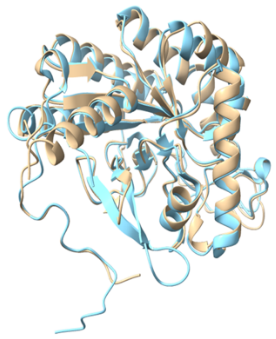

FIG S4. BLASTP search results, predicted structure, and predicted structural alignments for *L. pneumophila* protein 08520. (A) BLASTP results showing the similarity between strain 130b protein 08520 and the characterized PG deacetylases of *Burkholderia pseudomallei* and *Helicobacter pylori*. (B) Predicted 3-D structure of 08250, as determined by AlphaFold-3. The predicted structure is color-coded in accordance with the levels of confidence (i.e., pLDDT values) determined by the program (upper left). The protein's N-terminus and C-terminus are denoted as are the putative Zn-binding and active site. (C) Alignment of the predicted 08250 structure (in tan) with the known structure of the *B. pseudomallei* PG deacetylase (PDB 3S6O) (in turquoise), as analyzed using the DALI server. This statistical output for this alignment was: RMSD = 1.1, Z = 1.1, 44% identity.

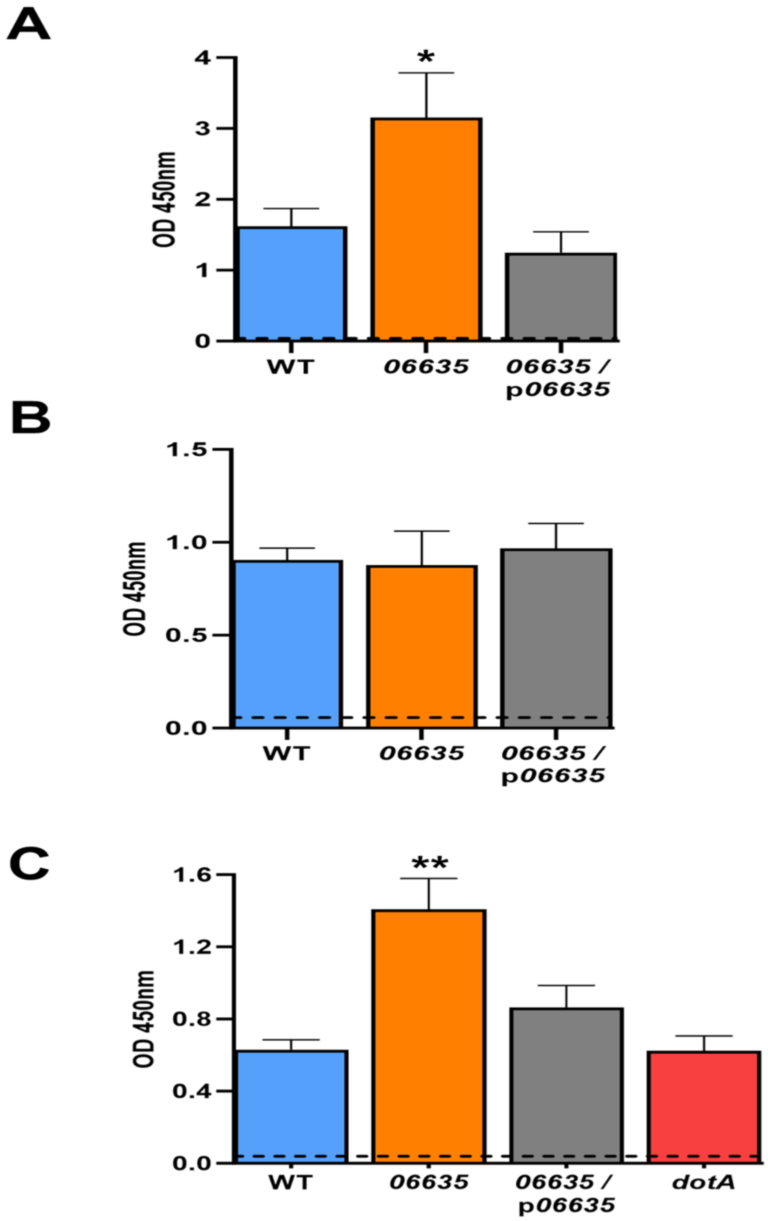

FIG S5. Further whole-cell ELISAs utilizing MAb 3/1, MAb 2-1, and anti-Lcl antibodies. (A - C) Following 3 d of growth on BCYE agar at 37°C, WT strain 130b (WT), the *06635* mutant NU486 (*06635*), NU486 containing plasmid-carried *06635* (*06635* / p*06635*), or the *dotA* mutant NU428 were subjected to whole cell ELISA (four technical replicates / strain) utilizing either the anti-LPS MAb 2-1 (A), anti-Lcl antibodies (B), or anti-LPS MAb 3/1 (C). Readings obtained from control wells containing no added bacteria are indicated by the horizontal dashed lines. In (A) and (C), the asterisks indicate greater antibody binding for the *06635* mutant relative to the other strains, *, $P < 0.05$; **, $P < 0.005$. The data presented are representative of the results obtained from three independent experiments.

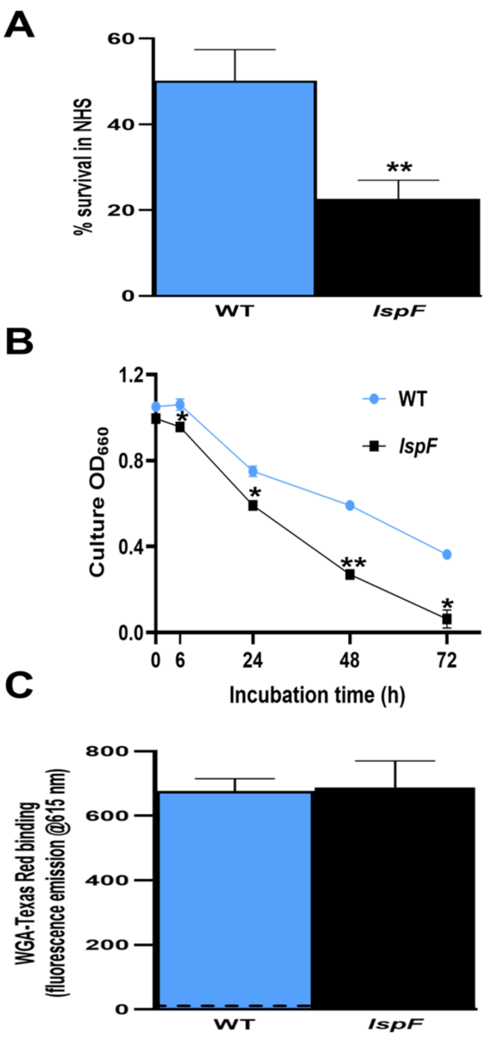

FIG S6. Effect of *lspF* mutation on serum-resistance, autoaggregation, and WGA-binding. (A) Following 3 d of growth on BCYE agar at 37°C, WT strain 130b (WT) or *lspF* mutant NU275 (*lspF*) were suspended in 90% NHS and statically incubated at 37°C. After 1 d, the percentages of surviving CFU were determined by plating. Data presented are the means and standard deviations from three technical replicates, and the asterisks indicate that the mutant behaved differently from WT, $P < 0.005$. (B) Following growth on BCYE agar, WT and *lspF* mutant were suspended in 10% BYE broth to an OD_{660} of ~1.0. Aliquots were added to tubes, and bacterial sedimentation at 37°C was assessed by measuring drops in the OD_{660} of the statically incubated suspensions. Data are the means and standard deviations from three technical replicates, and asterisks indicate that the mutant behaved differently from WT after $t = 0$, *, $P < 0.05$, **, $P < 0.005$. (C) Following growth in BYE broth at 37°C to early stationary phase, WT and *lspF* mutant were suspended in PBS with or without WGA-Texas Red. After 1 h at 37°C, the bacteria were washed and resuspended in PBS, and the amount of bound WGA-Texas Red quantified using a fluorimeter (excitation/emission at 595/615 nm). Readings from control wells containing no added bacteria are indicated by the horizontal dashed line. Data are the means and standard deviations from four technical replicates. The data presented are representative of the results obtained from two (A, C) or three (B) independent experiments

TABLE S1. BLASTP results indicating the presence of genes encoding 06635 and other putative T2SS substrates in *Legionella* species or *L. pneumophila* strains

A. Presence of genes encoding putative T2SS substrates within *Legionella* species

	Putative T2SS substrate*						
	**06635 (lpw20501)**	**11870 (lpw09611)**	**13770 (lpw05761**	**00420 (lpw00831)**	**08770 (lpw16101)**	**12705 (lpw07891)**	**06500 (lpw20771)**
L. adelaidensis	+	+	+	+	+	+	-**
L. anisa	+	+	+	+	+	+	+
L. antarctica	+	+	+	+	+	+	+
L. beliardensis	+	+	+	+	+	+	-
L. birminghamensis	+	+	+	+	+	+	+
L. bozemanae	+	+	+	+	+	+	+
L. brunensis	+	+	+	+	+	+	+
L. busanensis	+	+	+	+	+	+	+
L. cardiaca	+	+	+	+	+	+	+
L. cherrii	+	+	+	+	+	+	+
L. cincinnatiensis	+	+	+	+	+	+	+
L. clemsonensis	+	+	+	+	+	+	+
L. donaldsonii	+	+	+	-	+	+	+
L. drancourtii	+	+	+	+	+	+	+
L. drozanskii	+	+	-	+	+	+	+
L. dumoffii	+	+	+	+	+	+	+
L. erythra	+	+	+	+	+	+	+
L. fairfieldensis	+	+	+	+	+	+	-
L. fallonii	+	+	+	+	+	+	+
L. feeleii	+	+	+	+	+	+	+
L. geestiana	+	+	+	+	+	+	-
L. genomospecies	+	+	+	+	+	+	+
L. gormanii	+	+	+	+	+	+	+
L. gratiana	+	+	+	+	+	+	+
L. gresilensis	+	+	+	+	+	+	+
L. hackeliae	+	+	+	+	+	+	+
L. impletisoli	+	+	+	+	+	-	+
L. israelensis	+	+	+	+	+	+	+
L. jamestowniensis	+	+	+	+	+	+	+
L. jordanis	+	+	+	+	+	+	-
L. lansingensis	+	+	+	+	+	+	+
L. londiniensis	+	+	+	+	+	-	+
L. longbeachae	+	+	+	+	+	+	+
L. lytica	+	+	+	+	+	+	+
L. maceachernii	+	+	+	+	-	+	-
L. maioricensis	+	+	+	+	+	+	+
L. massiliensis	+	+	+	+	+	+	+
L. micdadei	+	+	+	+	+	+	-

L. moravica	+	+	+	+	+	+	+
L. nagasakiensis	+	+	+	+	+	+	+
L. nautarum	+	+	+	+	+	+	+
L. norrlandica	+	+	+	+	+	+	+
L. oakridgensis	+	+	+	+	+	+	+
L. parisiensis	+	+	+	+	+	+	+
L. pneumophila	+	+	+	+	+	+	+
L. polyplacis	-	-	-	+	-	-	-
L. qingyii	+	+	+	+	+	+	+
L. quateirensis	+	+	+	+	+	+	+
L. quinlivanii	+	+	+	+	+	+	+
L. rowbothamii	+	+	+	+	+	+	+
L. rubrilucens	+	+	+	+	+	+	+
L. sainthelensi	+	+	+	+	+	+	+
L. santicrucis	+	+	+	+	+	+	+
L. saoudiensis	+	+	+	+	+	+	+
L. septentrionalis	+	+	+	-	+	+	+
L. shakespearei	+	+	+	+	+	+	+
L. spiritensis	+	+	+	+	+	+	+
L. steelei	+	+	+	+	-	+	+
L. steigerwaltii	+	+	+	+	+	+	+
L. taurinensis	+	+	+	+	+	+	+
L. tucsonensis	+	+	+	+	+	+	+
L. tunisiensis	+	+	+	+	+	+	+
L. wadsworthii	+	+	+	+	+	+	+
L. waltersii	+	+	+	+	+	+	+
L. worsleiensis	+	+	+	+	+	-	+
L. yabuuchiae	+	+	+	+	+	-	+
**% prevalence**	**98 (65/66)**	**98 (65/66)**	**97 (64/66)**	**97 (64/66)**	**95 (63/66)**	**92 (61/66)**	**87 (58/66)**

* Former gene designations appear in parentheses.
**A "-" notation means that the BLASTP search did not reveal a match with an *E* value less than 0.05.

B. Presence of 06635 in sequenced strains of *L. pneumophila*

Accession Number	Strain	% Identity*	Source
GCF_041734345.1	130b	100	clinical
GCF_000048645.1	Paris	99	clinical
GCF_000048665.1	Lens	99	clinical
GCF_000008485.1	Philadelphia-1	99	clinical
GCF_002002645.1	Sudbury	99	clinical
GCF_000404245.1	Thunder Bay	99	clinical
GCF_001886795.1	Detroit-1	99	clinical
GCF_000239175.1	ATCC 43290	98	clinical
GCF_000347615.1	LPE509	99	clinical
GCF_000306865.1	Lorraine	99	clinical
GCF_002002625.1	Mississauga	98	clinical
GCF_001592705.1	Toronto-2005	99	clinical

GCF_000092545.1	Corby	99	clinical
GCF_000586295.1	ATCC 33215	99	clinical
GCF_000092625.1	Alcoy	99	clinical
GCF_000586135.1	ATCC 43283	99	clinical
GCF_000586255.1	ATCC 33737	99	clinical
GCF_000465675.1	Leg01/11	99	clinical
GCA_015989285.1	AZ00029759	99	clinical
GCF_001753085.1	C9_S	99	clinical
GCF_001766275.1	FFI102	99	clinical
GCF_004170265.1	NMB001870	99	clinical
GCF_004169905.1	NMB001868	99	clinical
GCA_015986385.1	A19030476	99	clinical
GCA_015894685.1	D1169	99	clinical
GCA_015893395.1	D5035	99	clinical
GCF_002813735.1	D5945	99	clinical
GCF_002813715.1	D6026	99	clinical
GCF_900452685.1	NCTC12000	99	clinical
GCF_001549925.1	PtVF66/2014	99	clinical
GCA_015894625.1	D1405	99	clinical
GCA_015895145.1	D3294	99	clinical
GCA_015895365.1	D4700	99	clinical
GCA_015950945.1	F4455	99	environmental
GCA_001766295.1	FFI103	99	environmental
GCA_001766355.1	FFI329	99	environmental
GCA_015975845.1	Isolate 4368	99	environmental
GCA_015971365.1	Isolate 4788	99	environmental
GCA_015970645.1	Isolate 4825	99	environmental
GCA_015979325.1	LG51	99	environmental
GCA_000347615.1	LPE509	99	environmental
GCA_001583655.1	SH003	99	environmental
GCA_015943225.1	CL20-200363	98	environmental
GCA_001886835.1	Dallas-1E	99	environmental
GCA_003205115.1	GC03	99	environmental
GCA_003205045.1	GC04	99	environmental
GCA_003205035.1	GC05	98	environmental
GCA_015977605.1	Isolate 3969	99	environmental
GCA_015976285.1	Isolate 4343	99	environmental
GCA_015970705.1	Isolate 4809	99	environmental
GCA_015971125.1	Isolate 4826	99	environmental
GCA_900637585.1	NCTC12273	99	environmental
GCA_001677115.2	Pontiac	99	environmental
GCA_015946125.1	CL20-200126	99	environmental
GCA_016021295.1	CL12-200318	99	environmental
GCA_015943185.1	CL20-200376	99	environmental
GCA_017716275.1	SU18-MZ1	99	environmental
GCA_017676575.1	SU18-MZ2	99	environmental

* % identity relative to the 130b 06635

TABLE S2. Previously confirmed T2SS substrates detected in current proteomic analysis

ORF	Protein Product	Fold-increase in WT supernatants over *lspF* mutant supernatants
plcB	eukaryotic-like phospholipase C PlcB	310.0
plcA	eukaryotic-like phospholipase C PlcA	187.4
nttE	novel type two secreted protein NttE	126.8
lcl	collagen-like protein, Lcl	98.5
nttD	novel type two secreted protein NttE	87.1
nttB	novel type two secreted protein NttB	65.6
map	eukaryotic-like major acid phosphatase	58.2
nttG	novel type two secreted protein NttG, VirK-like protein	49.5
lapB	eukaryotic-like lys/arg aminopeptidase LapB	42.2
proA	neutral metalloproteinase, ProA	38.3
lapA	eukaryotic-like aminopeptidase LapA	35.2
legP	eukaryotic-like, astacin-like protease LegP	31.9
chiA	chitinase ChiA	25.6
celA	endoglucanase CelA	22.7
amiA	putative amidase AmiA	11.5
lipB	triacylglycerol lipase LipB	5.8
lirB	putative peptidyl proline *cis-trans*-isomerase LirB	5.6
nttF	novel type two secreted protein NttF	4.4
nttC	novel type two secreted protein NttC	3.8
lipA	monoacylglycerol lipase LipA	2.5

TABLE S3. Full set of supernatant proteins detected in the current proteomic analysis*

24-3326_All , Report created on 11/14/23	Protein IDs	Fasta headers	Fold change WT/Lspf (log2) Student's T-test Difference LFQ intensity_WT_LFQ intensity_LspF (average WT LFQ-average LspF LFQ)	-Log Student's T-test p-value LFQ intensity_WT_LFQ intensity_LspF	LFQ intensity (WT 1)
	Q5ZUY7	tr|Q5ZUY7|Q5ZUY7_LEGPH Neutral metalloproteinase OS=Legionella pneumophila subsp. pneumophila (strain Philadelphia 1 / ATCC 33152 / DSM 7513) OX=272624 GN=lasB PE=3 SV=1	12.83771233	4.257523787	5.942949
	Q5ZXC8	tr|Q5ZXC8|Q5ZXC8_LEGPH Choloylglycine hydrolase OS=Legionella pneumophila subsp. pneumophila (strain Philadelphia 1 / ATCC 33152 / DSM 7513) OX=272624 GN=lpg0804 PE=3 SV=1	9.248694467	4.754421168	8.082985
	Q5ZWW3	tr|Q5ZWW3|Q5ZWW3_LEGPH Ecto-ATP diphosphohydrolase II OS=Legionella pneumophila subsp. pneumophila (strain Philadelphia 1 / ATCC 33152 / DSM 7513) OX=272624 GN=lpg0971 PE=4 SV=1	9.001534667	4.553636475	5.093752
	Q5ZVI5	tr|Q5ZVI5|Q5ZVI5_LEGPH Phospholipase C OS=Legionella pneumophila subsp. pneumophila (strain Philadelphia 1 / ATCC 33152 / DSM 7513) OX=272624 GN=lpg1455 PE=4 SV=1	8.276250667	4.1458935	3.901754

* The entire listing appears in the attached Excel file, with identifiers linked to the ORF designations in the *L. pneumophila* strain Philadelphia-1 genome. Listed here, as examples, are the four proteins most abundant in WT vs. *lspF* mutant supernatant.

TABLE S4. BLASTP results indicating the relatedness of protein 06635 to proteins outside of the *Legionella* genus

A. Proteins (i.e., hypothetical putative polysaccharide deacetylases) most related to 06635 *

Species*	Class	Coverage	E value	% Identity	Accession
Coxiella burnetii	*γ-proteobacteria*	81	4E-79	50	WP_094308884.1
Agrobacterium cavarae	*α-proteobacteria*	84	8E-37	35	WP_312809571.1
Jannaschia aquimarina	*α-proteobacteria*	81	3E-36	33	WP_043918491.1
Dyadobacter psychrophilus	*cytophagia*	84	5E-36	36	WP_082212656.1
Spirosoma sordidisoli	*cytophagia*	81	2E-35	32	WP_129605977.1
Porifericola rhodea	*cytophagia*	85	3E-35	33	WP_302242646.1
Fibrella forsythia	*cytophagia*	81	3E-35	35	WP_207330447.1
Arsenicibacter rosenii	*cytophagia*	81	6E-35	33	WP_071502238.1
Spirosoma lacussanchae	*cytophagia*	86	7E-35	31	WP_138502709.1
Inquilinus limosus	*α-proteobacteria*	79	7E-35	35	WP_034835512.1
Dyadobacter chenhuakuii	*α-proteobacteria*	83	1E-34	36	WP_235163394.1
Fibrivirga algicola	*cytophagia*	81	2E-34	32	WP_166692522.1
Cytophaga hutchinsonii	*cytophagia*	89	3E-34	33	WP_011584100.1
Runella zeae	*cytophagia*	94	4E-34	35	WP_051211292.1
Catalinimonas niigatensis	*cytophagia*	88	4E-34	33	WP_302250602.1
Lacihabitans lacunae	*cytophagia*	88	6E-34	32	WP_379838707.1
Aquicella siphonis	*γ-proteobacteria*	87	1E-33	33	WP_148338638.1
Yoonia maricola	*α-proteobacteria*	89	2E-33	30	WP_100368942.1
Pseudaestuariivita rosea	*α-proteobacteria*	81	3E-33	32	WP_208352827.1
Fibrisoma limi	*cytophagia*	84	5E-33	30	WP_009282455.1
Litoribacter populi	*cytophagia*	92	2E-32	33	WP_143960277.1
Echinicola marina	*cytophagia*	88	3E-32	31	WP_226333575.1
Pseudoxanthomonas sacheonensis	*γ-proteobacteria*	82	4E-32	31	WP_310092515.1
Catalinimonas alkaloidigena	*γ-proteobacteria*	83	5E-32	33	WP_277476586.1
Stenotrophomonas maltophilia	*γ-proteobacteria*	81	5E-32	30	HDS1581581.1
Fulvivirga imtechensis	*cytophagia*	83	9E-32	33	WP_009583460.1
Pseudofulvimonas gallinarii	*γ-proteobacteria*	83	1E-31	30	WP_123522109.1
Aridibacter famidurans	*blastocatellia*	81	3E-31	33	MCO6512627.1
Jiulongibacter sediminis	*cytophagia*	92	3E-31	32	WP_055143973.1
Epibacterium ulvae	*α-proteobacteria*	80	5E-31	32	WP_157843997.1
Sulfuriferula thiophila	*β-proteobacteria*	92	5E-31	27	WP_124949682.1
Larkinella punicea	*cytophagia*	85	8E-31	30	WP_114407515.1
Thiobacillus thioparus	*β-proteobacteria*	86	9E-31	30	WP_040723387.1
Tenacibaculum sediminilitoris	*flavobacteria*	96	1E-30	33	WP_408024091.1

Arcticibacterium luteifluviistationis	*cytophagia*	90	1E-30	33	WP_229201270.1
Aureibaculum algae	*flavobacteria*	90	1E-30	30	WP_138950879.1
Novosphingobium terrae	*α-proteobacteria*	81	2E-30	32	WP_206237805.1
Nannocystis exedens	*polyangia*	82	2E-30	30	WP_170136400.1
Ekhidna lutea	*cytophagia*	91	2E-30	33	WP_089355586.1
Geothrix limicola	*holophagae*	70	2E-30	35	WP_285578160.1
Gracilimonas mengyeensis	*balneolia*	89	3E-30	30	WP_142455866.1
Emticicia oligotrophica	*cytophagia*	84	4E-30	32	WP_015029594.1
Flexithrix dorotheae	*cytophagia*	91	4E-30	31	WP_020532240.1
Nocardiopsis yanglingensis	*actinomycetes*	82	5E-30	30	WP_412704669.1
Persicitalea jodogahamensis	*cytophagia*	86	6E-30	32	WP_189563694.1
Aureibaculum flavum	*flavobacteria*	90	1E-29	30	WP_198841767.1
Nibrella saemangeumensis	*cytophagia*	85	3E-29	30	WP_345246789.1
Planktotalea frisia	*α-proteobacteria*	69	4E-29	35	WP_111445375.1
Luteimonas composti	*γ-proteobacteria*	81	4E-29	30	WP_280941760.1
Acetobacter indonesiensis	*α-proteobacteria*	90	8E-29	32	WP_237595144.1
Granulicella rosea	*terriglobia*	87	1E-28	33	WP_089406600.1
Lysobacter enzymogenes	*γ-proteobacteria*	84	1E-28	30	WP_250451366.1
Pontibacter silvestris	*cytophagia*	88	1E-28	30	WP_229962985.1
Steroidobacter agaridevorans	*γ-proteobacteria*	81	1E-28	32	WP_202624295.1
Sporocytophaga myxococcoides	*cytophagia*	86	2E-28	31	WP_028978398.1
Arenimonas oryziterrae	*γ-proteobacteria*	81	2E-28	31	WP_022967896.1
Longimicrobium terrae	*longimicrobiia*	82	2E-28	31	WP_338088122.1
Tunicatimonas pelagia	*cytophagia*	86	2E-28	30	WP_302203638.1
Caulobacter vibrioides	*α-proteobacteria*	87	5E-28	31	OYX04264.1
Lacihabitans soyangensis	*cytophagia*	85	5E-28	31	WP_255035565.1
Sandaracinobacteroides hominis	*α-proteobacteria*	88	1E-27	32	WP_199555135.1
Terracidiphilus gabretensis	*terriglobia*	88	2E-27	30	WP_082662228.1
Wenzhouxiangella sediminis	*γ-proteobacteria*	83	3E-27	33	WP_147307702.1
Casimicrobium huifangae	*β-proteobacteria*	95	4E-27	29	WP_238363571.1
Acidicapsa dinghuensis	*terriglobia*	80	1E-26	30	WP_263339423.1
Chondrinema littorale	*cytophagia*	93	2E-26	31	WP_284686403.1
Oleiagrimonas citrea	*γ-proteobacteria*	82	2E-26	28	WP_168609839.1
Telluribacter humicola	*cytophagia*	83	3E-26	30	WP_207507809.1
Rubrivirga marina	*rhodothermia*	82	4E-26	31	WP_095509784.1
Comamonas aquatilis	*β-proteobacteria*	91	4E-26	29	WP_415797022.1
Zoogloea dura	*β-proteobacteria*	74	4E-26	33	WP_169146987.1
Silvanigrella aquatica	*oligoflexia*	87	4E-26	31	WP_148696286.1
Sphingomonas psychrolutea	*α-proteobacteria*	86	5E-26	29	GGA37740.1

Leeia aquatica	*β-proteobacteria*	93	6E-26	28	WP_168877343.1
Bacteriovorax stolpii	*bacteriovoracia*	88	7E-26	32	WP_102244984.1
Asticcacaulis taihuensis	*α-proteobacteria*	80	8E-26	29	WP_090645019.1
Brevundimonas diminuta	*α-proteobacteria*	89	8E-26	31	WP_242077931.1
Acinetobacter amyesii	*γ-proteobacteria*	92	1E-25	29	WP_249734402.1
Pleionea sediminis	*γ-proteobacteria*	87	1E-25	28	WP_144393462.1
Flavobacterium microcysteis	*flavobacteria*	91	1E-25	33	WP_139998478.1
Peristeroidobacter agariperforans	*γ-proteobacteria*	83	2E-25	30	WP_129646951.1
Berkiella aquae	*γ-proteobacteria*	88	2E-25	31	MCS5712855.1

* For conciseness, only a single representative species per genus is listed. This list is also not exhaustive, as it does not include the many results that have *E* values between 2e-25 and borderline significance.

B. Relationships between 06635 and characterized CE-4 superfamily members

Protein	Species	% Coverage	E value	% Identity	Accession
Peptidoglycan deacetylase	*Streptococcus pneumoniae*	53	3e-10	27	AVN86399.1
Peptidoglycan deacetylase	*Bacillus cereus*	82	2e-09	24	BCD24491.1
Peptidoglycan deacetylase	*Mycobacterium tuberculosis*	35	3e-08	30	WP_003898725.1
Acetylxylan esterase	*Caldanaerobacter subterraneus*	81	9e-09	23	KKC30048.1

TABLE S5. Synthetic DNAs used for study

A. PCR primers

Name	Description	Sequence (5' to 3') *
CA1	06635 5'F	CAAAGGAGCAATTAAAAACACAGCACGTG
CA2	06635 5'R	GAAGCAGCTCCAGCCTACACACGGAAACTGAAAAGAAAGAAATTAATGAGCCTC
CA3	06635 3'F	TAAGGAGGATATTCATATGGGCGATTTCATGTTCTTCGGCAAAG
CA4	06635 3'R	GGAATCATCACCCTTGTTTCGATAAAAGCG
CA5	06635_KanF	GAGGCTCATTAATTTCTTTCTTTTCAGTTTCCGTGTGTAGGCTGGAGCTGCTTC
CA6	06635_KanR	CTTTGCCGAAGAACATGAAATCGCCCATATGAATATCCTCCTTA
CA7	11870 5'F	GCATGA**GCGGCCGC**GCCCTTTATTCTGTGTTATTGGTGTCTTTTT (NotI)
CA8	11870 5'R	GAAGCAGCTCCAGCCTACACAGAGCCAGGCCATGCTGTTGTCGGTAAT
CA9	11870 3'F	TAAGGAGGATATTCATATGAAATTAAAATAGGGTGTTTAACAAAATTTT
CA10	11870 3'R	GCATGA**GTCGAC**GCTGTCCATGCTTTGAGCACGACTTAT (SalI)
CA11	11870_KanF	ATTCTTAATAATAAATTATGAGGATTTCCTTGTGTAGGCTGGAGCTGCTTC
CA12	11870_KanR	AAAATTTTGTTAAACACCCTATTTTAATTTCATATGAATATCCTCCTTA
CA13	13770 5'F	GCATGA**GCGGCCGC**GCCACATCAATATGAGAGTCAGCCTGTAATTG (NotI)
CA14	13770 5'R	GAAGCAGCTCCAGCCTACACAGGAAGAAACTGGTAAAAAATGCGAGTAATCTGG
CA15	13770 3'F	TAAGGAGGATATTCATATGGGAGCCTAGAACAACAATTAATCGCCTTTG
CA16	13770 3'R	GCATGA**GTCGAC**GGAGACTCCAGCTGATATGGCTAGAG (SalI)
CA17	13770_KanF	CCAGATTACTCGCATTTTTTACCAGTTTCTTCCTGTGTAGGCTGGAGCTGCTTC
CA18	13770_KanR	CAAAGGCGATTAATTGTTGTTCTAGGCTCCCATATGAATATCCTCCTTA
CA19	08770 5'F	GCATGA**GCGGCCGC**GGTGAGTCATCAGGGTAACTCTCTACATCATTG (NotI)
CA20	08770 5'R	GAAGCAGCTCCAGCCTACACACACAAAATGAAGTTATACAGGAATCGGTTGC
CA21	08770 3'F	TAAGGAGGATATTCATATGGTCGCAAAAGCTGTGATTAACAGCGTTTTAG
CA22	08770 3'R	GCATGA**GTCGAC**CAATTCTGCCAGGATGACGCCAAAAC (SalI)
CA23	08770_KanF	GCAACCGATTCCTGTATAACTTCATTTTGTGTGTGTAGGCTGGAGCTGCTTC
CA24	08770_KanR	CTAAAACGCTGTTAATCACAGCTTTTGCGACCATATGAATATCCTCCTTA
CA25	00420 5'F	GCATGA**GCGGCCGC**CCACTCCTCCAATTAAACTGAATGCAACAAC (NotI)
CA26	00420 5'R	GAAGCAGCTCCAGCCTACACACGTAGCCATAGATTTGAGCTTCACTGTTTGCC
CA27	00420 3'F	TAAGGAGGATATTCATATGCACAACTAAATCCAAGAGCCCAGTGAAC
CA28	00420 3'R	GCATGA**GTCGAC**CCCATATTGCCAAATCAGAAACTCTTTTGGC (SalI)
CA29	00420_KanF	GGCAAACAGTGAAGCTCAAATCTATGGCTACGTGTGTAGGCTGGAGCTGCTTC
CA30	00420_KanR	GTTCACTGGGCTCTTGGATTTAGTTGTGCATATGAATATCCTCCTTA
CA31	12705 5'F	GCATGA**GCGGCCGC**AAGTCCAAATACCGGCAAACAGCATGG (NotI)
CA32	12705 5'R	GAAGCAGCTCCAGCCTACACAGAAATTTCCAAAGCAGTGTGGGGTAATTG
CA33	12705 3'F	TAAGGAGGATATTCATATGCATTTGTATCGGCATTGTTTATCTCCAA
CA34	12705 3'R	GCATGA**GTCGAC**GAATTGAAAATGCTCAGGGTTGCCAGG (SalI)
CA35	12705_KanF	CAATTACCCCACACTGCTTTGGAAATTTCTGTGTAGGCTGGAGCTGCTTC
CA36	12705_KanR	TTGGAGATAAACAATGCCGATACAAATGCATATGAATATCCTCCTTA
CA37	06500 5'F	GCATGA**GCGGCCGC**GAACCAATTTGTTGTCTCTGGAGTTACGCC (NotI)
CA38	06500 5'R	GAAGCAGCTCCAGCCTACACAGCAAGCGATAAAAGACTTAATCCCAATATTCC
CA39	06500 3'F	TAAGGAGGATATTCATATGCGTTGGATATGATGGCGTAGGCTGTTATTT
CA40	06500 3'R	GCATGA**GAATTC**GAAACTCCGATGGCCTGTATTCAGGATC (EcoRI)
CA41	06500_KanF	GGAATATTGGGATTAAGTCTTTTATCGCTTGCTGTGTAGGCTGGAGCTGCTTC
CA42	06500_KanR	AAATAACAGCCTACGCCATCATATCCAACGCATATGAATATCCTCCTTA
CA43	Kan Internal F	GGGCACAACAGACAATCGGC
CA44	Kan internal R	CTCTTCAGCAATATCACGGGTAGCC
CA45	IspF 5'F	GCATGA**GCGGCCGC**AGCTGTAGCTACTTCATGATGATGTGCTTC (NotI)
CA46	IspF 5'R	GAAGCAGCTCCAGCCTACACAATTCACTTTTAGCGCTTGATATTGGTAGGC
CA47	IspF 3'F	TAAGGAGGATATTCATATGGAGATTGGTAACTGGGATGACCACTCATAA
CA48	IspF 3'R	GCATGA**GTCGAC**CCTGCTTGTTTGGAACTGACTGAAATGGTT (SalI)
CA49	IspF_GentF	GCCTACCAATATCAAGCGCTAAAAGTGAATTGTGTAGGCTGGAGCTGCTTC
CA50	IspF_GentR	TTATGAGTGGTCATCCCAGTTACCAATCTCCATATGAATATCCTCCTTA
CA51	06635_pmmBGentF	GCATGA**GAGCTC**ATAACCCTTATGCATAACTCACGTTAATTT (SacI)
CA52	06635_pmmBGentR	GCATGA**CCCGGG**TTAAGATTGAGGCTCATTAATTTCTTTCTT (SmaI)
OR77	Vector Fwd	TCGGCTCGTATAATGTGTGG
OR78	Vector Rev	ACCGCTTCTGCGTTCTGATT
CA53	06635 Flag1_For	GCATGA**GGTACC**ATGTTAAAACGCTTACTCGGTGTAGCATCATT (KpnI)
CA54	06635 Flag1_Rev	CGTACT**GGTACC**TTACTTGTCGTCATCGTCTTTGTAGTCAGATTGAGGCTCATTAATTTCTTTCTTTTCAGTTTCCG (KpnI)
CA55	06635 Flag2_For	GCATGA**GAGCTC**ATGTTAAAACGCTTACTCGGTGTAGCATCATT (SacI)
CA56	06635 Flag2_Rev	CGTACT**GGTACC**TTACTTGTCGTCATCGTCTTTGTAGTCAGATTGAGGCTCATTAATT (KpnI)

* Nucleotides in bold indicate the locations of restriction enzyme sites that facilitated cloning. The corresponding enzymes are listed to the right of the sequence and in parentheses.

B. *06635* gene used for cloning

**Sequence (5’ to 3’)**
CCATGGTGGAAGATCATGAAATTGCCATCACCATTGATGATCTGCCGTTTGTTGGTAGCGGCACCAATACACCGGGTAATCTGAAACGTACCCAAGAACGTTTTATGGCCATTGTTAATA<br>CCCTGGTGGATAATCAGGTTCCGGCAACCGGTTTTGCAATTGGTGGTGCAATTGCCAAAAATGAATGGGAACTGCTGGAAATTTTTCGCAATCAGGGTTTTAGCATTGGCAACCATACCT<br>ATAAACATCGTAGCCTGAATAGCATGACCGCAGAAAACTATATTGCCGATATTGAAAAAGCCGATACCGTTCTGAGTCCGGTTATGACCGAACCGAAATACTTTCGTTATCCGTATCTGG<br>CCGAAGGTAGCGGTGAAAAGAAACAGAAAGTTCATGAATGGCTGGCAGCACATCAGTATACCATTGCACCGGTTACCATTGATAGCAAAGATTATGAATTTAACGCCCAGTTTTATCGCA<br>TTCCGTATCGTCAGCGTCCGCAGCGTCTGGCACAGTTTAAAAAGCGTTATCTGGCATTTATTTGGCAGCAGACCCTGCGTGCCGAAAAGAAAGTTAAAAAGGTTGAAGGTCAGCCGGTT<br>AAACATATTCTGCTGATTCATGCCAATCTGATTAATAGCCTGTGTCTGGCCGATATCATTGAAATGTATCGTAGCAACGGCTACAAATTCATTACCCTGCAAGAAGCACTGAAAGGTAATA<br>CCGCAACACCGGTTAATGATAGCAGCCCGACCGAAGCGCTGAAAAGCGAAACCGAGAAGAAAGAAATTAATGAACCGCAGAGCCTCGAG
